# Redefining media blending mathematically: a systematic approach for screening of medium components

**DOI:** 10.1007/s00253-025-13594-z

**Published:** 2025-12-08

**Authors:** Hirotaka Kuroda, Kazuya Sorada, Noriko Yamano-Adachi, Takeshi Omasa

**Affiliations:** 1 Graduate School of Engineering, Osaka University, 2-1 Yamadaoka, Suita, Osaka, 565-0871 Japan; 2 Shimadzu Corp, 1 Nishinokyo Kuwabara, Nakagyo-Ku, Kyoto, 604-8511 Japan; 3 Shimadzu Analytical Innovation Research Laboratory, Osaka University, 2-1 Yamadaoka, Suita, Osaka, 565-0871 Japan; 4 Institute for Open and Transdisciplinary Research Initiatives, Osaka University, 2-1 Yamadaoka, Suita, Osaka, 565-0871 Japan; 5 Manufacturing Technology Association of Biologics (MAB), 7-1-49 Minatojima-Minamimachi, Chuo, Kobe, Hyogo 650-0047 Japan

**Keywords:** Media blending, Medium component screening, Chemically defined media, Mammalian cell culture, New methodology

## Abstract

**Abstract:**

Biopharmaceuticals, such as antibody therapeutics, are produced by culturing mammalian cells with chemically defined media that consist of more than 50 synthesized components. The screening of medium components related to culture performance and the subsequent optimization of the composition are required in the development of new modalities, host cells, and culture methods. Screening the components to be optimized is typically labor-intensive. The easiest approach is media blending, which creates variations in the concentrations of the components with only liquid mixing. However, a workflow for systematically determining experimental conditions (i.e., how to blend media) has not been established. Therefore, we reassessed media blending from a mathematical perspective and proposed a workflow for the first time. In the workflow, we evaluated the use of a commercially available chemically defined media to maximize simplicity and applicability. From a mathematical perspective, we clarified that multicollinearity is an inevitable challenge in both experimental design and its analysis. Under the constraint, we showed that one of the most appropriate experimental conditions could be systematically calculated and selected by applying D-optimal design focusing on the principal components. We performed a case study of cell culture to screen medium components under 120 experimental conditions using 11 chemically defined media designed for Chinese Hamster ovary cells. The case study provided a reasonable set of components that explained the variance in viable cell concentrations, which range from 5.8 to 19.4 (× 10^6^) cells/mL. Finally, our mathematical redefinition also enabled the design of a dedicated media set for media blending.

**Key points:**

• *The constraints in media blending were clearly explained.*

• *A systematic workflow from blending design to analysis was proposed.*

• *The workflow also enabled the design of a dedicated media set for media blending.*

**Supplementary information:**

The online version contains supplementary material available at 10.1007/s00253-025-13594-z.

## Introduction

Mammalian cell culturing is an indispensable process for producing active biopharmaceutical ingredients, such as antibody therapeutics and gene therapeutics (O’Flaherty et al. 2020). It is also essential for using the cells as final products, including cellular food and regenerative medicine (Ozawa et al. 2023; Martins et al. 2024). Historically, medium composition has been studied and discussed for the improvement of biomass accumulation and the amount of titer because the cells consume the medium components for their own growth and production of the target product (Landauer 2014; Ritacco et al. 2018). Chemically defined media (CDM) that consist only of synthesized components are preferred when the final product is intended for human use because of the prevention of the risk of viral infection and the ease of the management of raw materials. For example, many basal and feed CDM have been developed for the culture of Chinese hamster ovary (CHO) cells (Reinhart et al. 2015). Although the composition of CDM developed by suppliers is sufficiently sophisticated to achieve specific applications, the individual optimization of the composition is still required. In various stages of culture process development, such as cell line modification and culture methods (batch, fed-batch, and perfusion culture), the identification of the optimal medium composition has been explored (Wurm 2004; Galbraith et al. 2018). Furthermore, when new host cells or modalities are used, the development of a completely new composition may need to be considered.

CDM commonly consists of more than 50 components (Xu et al. 2018; Galbraith et al. 2018; Cordova et al. 2023). The simultaneous optimization of all components is difficult because the increase in the number of targeted components to be optimized expands the search space exponentially. Additionally, the number of implementations of cell culture at one time is limited, and the process requires significant cost and time. Therefore, the optimization of the composition is commonly conducted by focusing on specific components. A review of medium optimization indicated that the average number of conditions per experimental batch was 27 and the average number of components to be optimized was 8.1 (Zhou et al. 2023). The components to be optimized are screened through experimental evaluation to determine those closely related to culture performance. The most common approaches are one factor at a time (OFAT) and factorial design-based methods (Combe and Sokolenko 2021). OFAT is performed by changing the concentration of only one variable, whereas factorial design is performed by changing several variables simultaneously. The selection of the approach depends on the number of components to be evaluated and the need to take into account interactions (Yasui et al. 2021; Hashizume and Ying 2024). For example, the Plackett–Burman design (Plackett and Burman 1946) is suitable for screening multiple variables generally, although interactions are disturbed. When OFAT and factorial design are applied to screening medium components, these approaches typically require the laborious preparation of concentrated solutions of the individual components. The preparation of concentrated solutions seems to be a simple operation, but it requires using solvents with different pH values that depend on the components because of solubility considerations, which necessitates pH adjustment of the medium after the supplementation of the components. This is quite an arduous operation, particularly when the number of components to be screened increases, and it is unsuitable for the automation of experiments.

Media blending is an easy approach that creates variations in the concentrations of the components to be evaluated with only the pipette operation of liquid mixing. Jordan and Rouiller et al. evaluated medium components related to cell growth and titer in antibody-producing CHO cells by designing the original media used for blending, referred to as the “mother media” in this study (Jordan et al. 2013; Rouiller et al. 2013). However, media blending has not been widely adopted because a workflow for systematically determining the experimental conditions (i.e., how to blend the mother media) has not been established. The establishment of a systematic workflow requires handling the process mathematically. Additionally, considering the issue mathematically will enable a structured evaluation of the limitations and applicability of media blending. Therefore, in this study, we reassessed media blending from a mathematical perspective and proposed a workflow for the initial screening of the components to be optimized. In the development of the workflow, it is important that it is user-friendly and immediately usable for any case by all researchers. Thus, we notably used commercially available CDM as mother media in the evaluation. There are two main advantages to using CDM as mother media. The first is that the design and preparation of the mother media is not required. This point is simple but important because the preparation of the mother media is the only laborious step in the media blending approach. For simplicity, material candidates include commercially available stock solutions for each component category (e.g., amino acids, vitamins, and trace salts) used by Biederman et al. (2022) in modular blending; however, we consider the following second advantage: The selection of CDM that meets the intended purpose allows researchers to reference empirically adjusted concentrations. This enables the straightforward incorporation of these concentration ranges into experimental conditions, even for the high concentrations in production and feed CDM for biopharmaceuticals. Although the range of concentrations to be tested depends on the chosen CDM, components with small variance among products can be considered to reflect an empirical consensus.

To summarize the key points, the objective of this study is to develop a workflow that can systematically determine experimental conditions by reassessing media blending from a mathematical perspective. In the development of the workflow, we evaluate the use of commercially available CDM to maximize simplicity and applicability, thereby ensuring it can be widely used by all researchers.

## Materials and methods

**Fig. 1:**
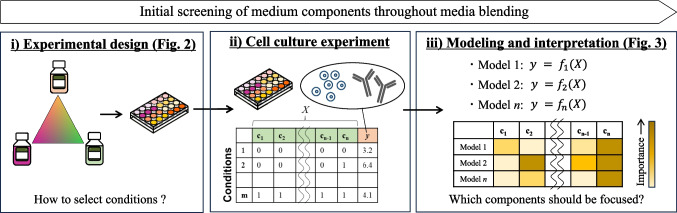
Overview of the entire process for the initial screening of medium components through media blending

Initial screening of the medium components involves constructing a model y=f(X) for culture performance y (e.g., viable cell concentration (VCC) or product concentrations) using X, in which the medium components are variables, and then interpreting the model to estimate the medium components that are most closely related to y. Thus, a methodology that carries out this process effectively and logically is important. Our proposed workflow for the initial screening of medium components through media blending with commercially available CDM consists of the following three steps: (ⅰ) experimental design, (ⅱ) cell culture experiments, and (ⅲ) regression modeling and its interpretation (Fig. 1a).

### Step (ⅰ)−1: Experimental design (theory)

#### Definition of the matrices in media blending

First, we define the composition of the mother media using matrix A, defined as follows:

1 $$A \in {\mathbb{R}}^{m \times d} ,\quad a_{ij} \ge 0\;(\forall i,j)$$

$$where\left\{ {\begin{array}{*{20}l} {m:{\text{the number of mother media}},} \hfill \\ {d:{\text{the number of medium components}}.} \hfill \\ \end{array} } \right.$$

$$A = \left[ {\begin{array}{*{20}c} {a_{11} } & {a_{12} } & \cdots & {a_{1d} } \\ {a_{21} } & {a_{22} } & \cdots & {a_{2d} } \\ \vdots & \vdots & \ddots & \vdots \\ {a_{m1} } & {a_{m2} } & \cdots & {a_{md} } \\ \end{array} } \right]$$

Next, we define the matrix D, which represents the dispensing volumes of the mother media for all possible blending combinations, as follows:

2 $$D \in {\mathbb{N}}^{k \times m}$$

$$where\left\{ {\begin{array}{*{20}l} {k:{\text{the number of combinations for blending mother media}},} \hfill \\ {m:{\text{the number of mother media}}.} \hfill \\ \end{array} } \right.$$

**Fig. 2:**
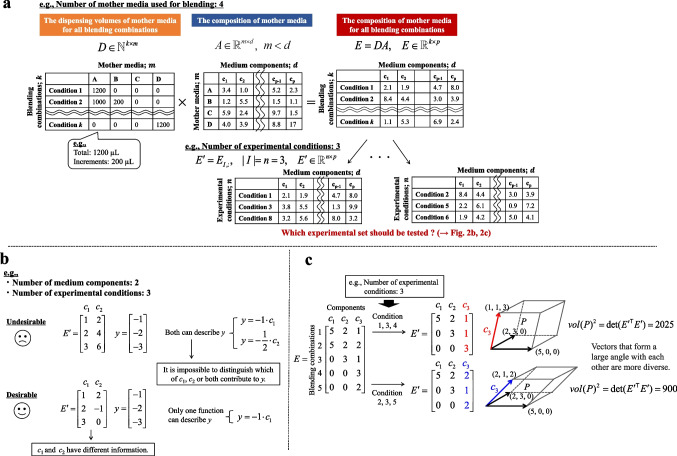

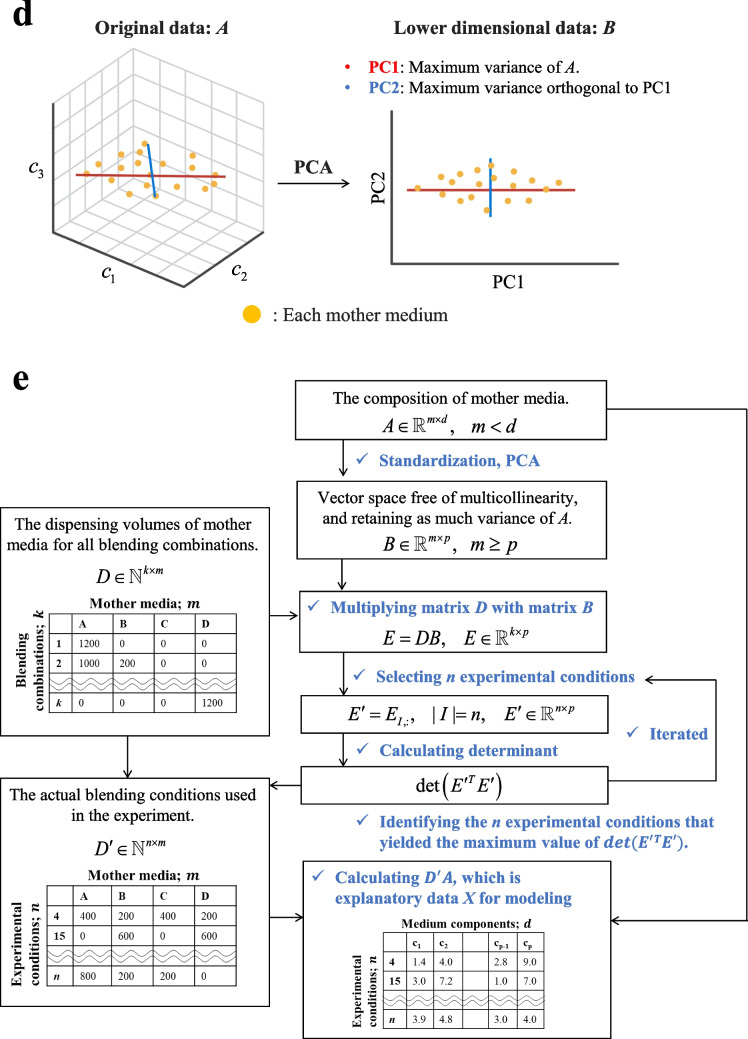
Systematic experimental design for the initial screening of medium components through media blending. **a** Initial definition of the matrices in media blending. To illustrate the general concept of experimental design in media blending, a simplified example is shown with four mother media and three final experimental conditions. **b** The desired situation in the experimental design for regression modeling, particularly for the aim of model interpretation. A simplified example is shown with two medium components and three final experimental conditions. This example illustrates that when there is high correlation between medium components (multicollinearity), it becomes challenging to determine which component is related to *y*. **c** Geometric illustration of selecting experimental conditions that minimize intervariable correlation (multicollinearity). In this simplified example, three experimental conditions are chosen from five options. Choosing conditions 1, 3, and 4 results in column vectors $c_{1}$, $c_{2}$, and $c_{3}$, which are oriented in more diverse directions than those when conditions 2, 3, and 5 are selected, thereby creating a larger vector space. Maximizing the volume of the space (parallelepiped) is equivalent to maximizing the diversity of the column vector directions. **d** Illustration of dimensionality reduction using principal component analysis (PCA). The original three-dimensional space defined by the concentrations of media components $c_{1}$, $c_{2}$, and $c_{3}$ for each mother medium is projected onto a two-dimensional space. The red line corresponds to the direction with the maximum variance, which is the 1 st principal component. The blue line, which is the vector orthogonal to the 1 st principal component with the maximum variance is the 2nd principal component. **e** Flowchart of our proposed systematic experimental design for the initial screening of medium components through media blending. Blue text represents execution steps, whereas black text indicates matrices. P, parallelepiped; PC, principal component

By multiplying matrices D and A, we obtain matrix E, which gives the composition of each medium component in all blending combinations (Fig. 2a):

3 $$E = DA,\quad E \in {\mathbb{R}}^{k \times d}$$

Given the limited number of conditions per experimental batch for a cell culture, it is impractical to conduct experiments for all possible combinations. Therefore, a specified number of experimental conditions, denoted by n, is extracted from the matrix E; that is,

4 $$E^{\prime} = E_{I,:} ,\quad |I| = n,\quad E^{\prime} \in {\mathbb{R}}^{n \times d}$$

$${\text{where }}n{\text{ is the number of experimental conditions}}{.}$$

#### Definition of the direction of experimental design

As mentioned above, the purpose of the initial screening is achieved by modeling y=f(X) and interpreting the model. In the context of media blending, the matrix $E^{\prime}$ in Eq. (4) corresponds to X in the model. If the columns of $E^{\prime}$ (each representing a different medium component) essentially provide the same information, it become impossible to distinguish which medium components contribute to y when constructing the model (Fig. 2b). This situation, in which the columns can be expressed as a linear combination of the other columns, is called “multicollinearity.” Therefore, in the experimental design step for the screening study, it is desirable to maximize the diversity of the column vectors of $E^{\prime}$ when extracting n rows from E. Geometrically, this means maximizing the volume of a d-dimensional parallelepiped formed by the standardized column vectors ${\mathbf{e^{\prime}}}_{1} ,{\mathbf{e^{\prime}}}_{2} , \ldots ,{\mathbf{e^{\prime}}}_{d}$ of $E^{\prime}$. In the three-dimensional case, a parallelepiped can be schematically represented as in Fig. 2c, where the volume enclosed by the column vectors corresponds to the volume of the parallelepiped. Clearly, the volume increases as the column vectors diverge in direction. Denoting the parallelepiped as P, its volume is defined as follows.

5 $$vol\left(P\right)=\sqrt{\det\left(E^TE'\right)}$$

Thus, maximizing the volume of parallelepiped is equivalent to solving the following expression.

6 $$\max\;\det\;\left(E'^TE'\right)$$

In the context of design of experiment (DoE), solving $\left(E'^T\;E'\right)$ is the D-optimal design problem. A wide range of methods can be used for DoE, including factorial design-based approaches (e.g., full factorial, fractional factorial, and Plackett–Burman design) as well as methods such as Latin hypercube sampling. However, the methods that assume that the values for the explanatory variables are independently specified, such as factorial design and Latin hypercube sampling, cannot be used in the media blending context. This is because the explanatory variables (i.e., the medium components in this study) are embedded within the manipulated variables (i.e., the mother media). Moreover, because the mother media themselves are predetermined (i.e., observational data), D-optimal design cannot be replaced by these DoE methods. Hence, we believe that solving for $\left(E'^T\;E'\right)$ (D-optimal design) is the most reasonable experimental design approach for the purpose of screening medium components.

#### Mathematical constraint in media blending

To state the conclusion first, the mathematical constraint in the experimental design for media blending is that $\left(E'^T\;E'\right)$ always becomes zero. In other words, it is impossible to implement the D-optimal design principle in the standard way, as described above. This issue arises from the rank deficiency of matrix $E'^T\;E'$. The rank of a matrix is defined as the maximum number of linearly independent rows or columns; therefore, the rank is always less than or equal to the smaller of the two. Rank deficiency occurs when the rank of a matrix is smaller than the number of rows or columns (whichever is smaller). To clarify why matrix $E'^T\;E'$ is rank deficient, we explain each step of the process that causes the rank deficiency. First, we consider the rank of matrix $A \in {\mathbb{R}}^{m \times d}$. As stated in the Introduction, the main advantage of media blending is the simplicity of its experimental procedures. Therefore, it is generally assumed that the number of mother media is less than the number of medium components (which is greater than 50), implying that m<d. Thus, the rank of the matrix A satisfies.

7 $${\mathrm{rank}}(A) \le m < d.$$

Next, for matrix D, since the number of all blending combinations k is assumed to be larger than the number of mother media m, the rank of matrix D satisfies

8 $${\mathrm{rank}} (D) \le m < k.$$

Therefore, the rank of the matrix E(where E=DA) is given by

9 $${\mathrm{rank}} (E) = {\mathrm{rank}} (DA) \le \min ({\mathrm{rank}} (D),{\mathrm{rank}} (A)) \le m.$$

Since matrix $E^{\prime}$ is obtained by extracting n rows (the specified number of experimental conditions) from matrix E, it follows that

10 $${\mathrm{rank}} (E^{\prime}) \le \left\{ {\begin{array}{*{20}l} {m,} \hfill & {{\text{if }}m < n,} \hfill \\ {n,} \hfill & {{\text{if }}n < m.} \hfill \\ \end{array} } \right.$$

Consequently, the rank of $E'^T\;E'$ is also

11 $$\begin{array}{c}\mathrm{rank}\;\left(E'^T\;E'\right)\leq\left\{\begin{array}{lc}m,&\mathrm{if}\;m<n,\\n,&\mathrm{if}\;n<m.\end{array}\right.\\\therefore\;\mathrm{rank}\;\left(E'^T\;E'\right)\leq m.\end{array}$$

As indicated in Eq. (4), we have $E'^T\;E'\;\in\;\mathbb{R}^{d\times d}$. However, since m<d, matrix $E'^T\;E'$ is rank deficient with respect to its dimension d. In a rank-deficient case, the determinant of the matrix is zero; hence,

12 $$det\;\left(E'^T\;E'\right)=0.$$

For further details on this derivation, please refer to Supplemental Note S1. Since $\max\;\det\;\left(E'^T\;E'\right)$ is always zero, the approach of directly solving the determinant for matrix $E'^T\;E'$, which is often a reasonable experimental design strategy, cannot be used. This is the mathematical constraint in the experimental design of media blending.

#### Reasonable solutions

On the basis of the derivation so far, the fundamental cause for $\det\;\left(E'^T\;E'\right)=0$ lies in the fact that the number of mother media m is smaller than the number of medium components d in matrix A. However, considering the advantage of the simplicity of media blending, it is necessary to permit m<d as a premise. Because rank deficiency inevitably leads to multicollinearity, allowing m<d implies tolerating partial multicollinearity. Furthermore, since matrix A consist of observational data from commercially available CDM, there exists the possibility of multicollinearity even if m≥d. Thus, it is necessary to select experimental conditions that minimize redundancy among the column vectors of $E^{\prime}$ under partial multicollinearity.

To overcome the mathematical constraint, it is necessary to reduce the dimensionality of matrix A into a vector space of at most m dimensions that is free of multicollinearity. It is important to consider the method used to achieve this. Possible methods for achieving such dimensionality reduction include variable filtering, which removes variables with low information based on metrics such as the variance inflation factor (VIF) or correlation coefficients, and principal component analysis (PCA), which reduces the number of dimensions while retaining as much information as possible in a space of linearly independent column vectors.

Among the possible methods, we took into account a characteristic feature of our study—the use of commercially available CDM as mother media—when selecting the most appropriate method. Commercially available CDM are pre-formulated products, meaning that their composition cannot be freely manipulated. The variability among CDM differs by component; some components are present at consistent levels across all products, while others exhibit considerable variability in different products. Under the basic assumption that differences in culture performances arise from variability in the composition of the mother media, we reasoned that it is important to reduce dimensionality in a manner that retains as much variance as possible. Accordingly, we pursued dimensionality reduction that achieves two objectives: reducing matrix A to a vector space free of multicollinearity and minimizing the loss of the variance.

The dimensionality reduction method that achieves these two objectives is formulated and explained in Supplemental Note S2. In brief, these objectives are achieved by performing repeated orthogonal projection onto the axis of maximum variance on the standardized matrix A. Here, this procedure corresponds exactly to the operation performed by PCA (Fig. 2d). In PCA, the original data space is reduced to a subspace formed by axes called principal components. Because the principal components are orthogonal, PCA can eliminate the redundant information (multicollinearity) that can obscure factor relationships in high-dimensional data. Furthermore, by selecting the directions of maximum variance first, PCA retains a substantial portion of the original data’s total variance. By mapping the original matrix $A^{\prime}$ onto these principal components ${\mathbf{v}}_{1} ,{\mathbf{v}}_{2} , \ldots ,{\mathbf{v}}_{p}$, the data is reduced to a p-dimensional space (p≤m) represented as follows:

13 $$B = A^{\prime}{\mkern 1mu} V_{p} ,\quad V_{p} = [{\mathbf{v}}_{1} ,\;{\mathbf{v}}_{2} ,\; \ldots ,\;{\mathbf{v}}_{p} ].$$

Therefore, given the two objectives—that is, to retain as much variance in the medium composition as possible and to reduce the dimensionality without multicollinearity—PCA is clearly the most appropriate approach.

We denote the matrix after dimensionality reduction by $B \in {\mathbb{R}}^{m \times p}$. Accordingly, matrix E, originally defined in Eq. (3), can be revised as

14 $$E = DB,\quad E \in {\mathbb{R}}^{k \times p} .$$

That is, E represents the locations in p-dimensional space (obtained after PCA) for each blending combination. From E, n rows are selected to construct $E^{\prime}$, as follows:

15 $$\begin{gathered} E^{\prime} = E_{I,:} ,\quad |I| = n,\quad E^{\prime} \in {\mathbb{R}}^{n \times p} \\ {\text{where }}n{\text{ is the number of experimental conditions}}{.} \\ \end{gathered}$$

Since matrix $E'^T\;E'$ is no longer rank deficient, the maximization of $\det\;\left(E'^T\;E'\right)$ becomes meaningful. When we employ $D^{\prime} \in {\mathbb{R}}^{n \times m}$, which assumes that n conditions have been selected from matrix D, matrix $E^{\prime}$ can be decomposed based on Eqs. (13) and (14) as follows:

16 $$E^\prime = D^\prime B = D^\prime (A^{\prime}V_{p} ) = (D^\prime A^{\prime})V_{p}$$

Thus,

17 $$E'^T\;E'=\left(D'A'V'_p\right)^T\;\left(D'A'V'_p\right)=V_p^TA'^T\left(D'\right)^T\;D'A'V_p.$$

Since $V_{p}$ is an orthonormal basis for the p-dimensional space, $E'^T\;E'$ is the matrix that expresses $A'^T\;D'^T\;D'A'$ within that subspace. Consequently, solving $\max\;\det\;\left(E'^T\;E'\right)$ is equivalent to mitigating multicollinearity in the non-redundant portion of $D^{\prime}A^{\prime}$. Since $D^{\prime}A^{\prime}$ corresponds to a matrix obtained by extracting n rows from DA (Eq. (3)), ensuring that the non-redundant portion of $D^{\prime}A^{\prime}$ maintains as much diversity as possible is consistent with the original problem to be solved.

Therefore, in a scenario where matrix A (i.e., a composition of mother media) is rank deficient, we propose the approach of employing PCA to form a p-dimensional space that retains as much of the variance A as possible, and then selecting experimental conditions that maximize $\det\;\left(E'^T\;E'\right)$ (i.e., D-optimal design). This approach helps reduce the undesirable intervariable correlations (multicollinearity) in the experimental conditions, thus aiding the interpretation of the regression model of the relationship between X (the medium components) and culture performance y. The proposed workflow represents the first systematic experimental design for the initial screening of medium components throughout media blending. This framework is presented in Fig. 2e.

### Step (ⅰ)−2: Experimental design (case study)

#### Chemically defined media

The CDM used in this study were 1X CD CHO (Thermo Fisher Scientific, Waltham, MA, USA, Cat# 10743-011); CH400AZ (GMEP, Fukuoka, Japan, Cat# CH400AZ-0005); HyClone™ ActiPro™ (Cytiva, Marlborough, MA, USA, Cat# SH31039.02); TC-42 w/o GF (Sartorius, Göttingen, Germany, Cat# 511-0001); CHOlean (Sartorius, Göttingen, Germany, Cat# 1140-0001); 4Cell® SmartCHO Stock & Adaptation Medium (Sartorius, Göttingen, Germany, Cat# CFP3FB1101); 4Cell® SmartCHO Media System Production Medium (Sartorius, Göttingen, Germany, Cat# CFP3FB2102); Ham’s F-12 Nutrient Mix (Thermo Fisher Scientific, Waltham, MA, USA, Cat# CFP3FB2102); RPMI 1640 (Thermo Fisher Scientific, Waltham, MA, USA, Cat# 11875-093); CD FortiCHO™ (Thermo Fisher Scientific, Waltham, MA, USA, Cat# A11483-01); CD OptiCHO™ (Thermo Fisher Scientific, Waltham, MA, USA, Cat# 12681-011); and EX-CELL® CD CHO Fusion (Sigma-Aldrich, St. Louis, MO, USA, Cat# 14365C-1000ML). EX-CELL® CD CHO Fusion is the medium to which the cells were originally acclimated and was used as the common medium (Fig. 5a). The other 11 protein-free media were used as mother media.

#### Media analysis using mass spectrometry

Since the composition of the CDM used in this study was mostly undisclosed, the components of the CDM were analyzed using triple quadrupole liquid chromatograph mass spectrometry (LC-MS/MS) and inductively coupled plasma mass spectrometry (ICP-MS).

For the LC-MS/MS analysis, the mother media were mixed with acetonitrile (FUJIFILM Wako Pure Chemical, Osaka, Japan, Cat# 018-19853), followed by centrifugation at 15,000 rpm for 15 min at room temperature. The supernatants from the centrifugation were collected and diluted tenfold with ultrapure water. The samples were then analyzed using the LCMS-8060NX (Shimadzu Corp., Kyoto, Japan). LC/MS/MS Method Package for Cell Culture Profiling Ver. 3 (Shimadzu Corp., Kyoto, Japan), which is tailored for cell culture samples and targets a total of 144 components (amino acids: 72, nucleic acids: 32, vitamins: 18, sugars: 4, and other components: 18), was used for the analytical condition. For the ICP-MS analysis, the mother media were diluted with 1% nitric acid (FUJIFILM Wako Pure Chemical, Osaka, Japan, Cat# 140-10045) to prepare the samples. The dilution ratios were 10-fold for trace metal ions (Al, Ba, Br, Ca, Cd, Co, Cu, Fe, Li, Mg, Mn, Mo, Rb, Se, Sn, Sr, Ti, V, and Zn) and 1000-fold for high-concentration metal ions (K, Na, and P). These metal ions were analyzed using the ICPMS-2030 (Shimadzu Corp., Kyoto, Japan) under the conditions listed in Supplemental Table S1.

#### Breakdown of each matrix

Here, in the case study, the breakdown of the matrices defined in the “Experimental design (theory)” section is presented.

First, matrix A, representing the composition of mother media as described in Eq. (1), was arranged such that its rows correspond to the various mother media types and its columns to the medium components, with each element representing the analytical value of a medium component determined by mass spectrometry.

Matrix A was then projected onto a low-dimensional orthogonal space that retains as much variance as possible via PCA, as shown in Eq. (13). So that each medium component had the same scale, matrix A was first standardized for each medium component to form $A^\prime$, and then PCA was applied to obtain matrix B. The dimensionality of B was chosen such that 99% of the variance in $A^\prime$ was retained.

Matrix D, which represents the dispensing volumes of the mother media for all possible blending combinations (Eq. (2)), was set up as follows in this case study. To avoid excessive experimental complexity, up to 6 of the 11 mother media were selected and blended in increments of 200 µL to yield a total volume of 1200 µL. Thus, the number of blending combinations (i.e., number of rows) was $$\left( \begin{gathered} 11 + 6 - 1 \\ 6 \\ \end{gathered} \right) = 8008$$.

According to Eq. (14), matrix E was derived as the product of matrices D and B. Matrix E indicates the locations in p-dimensional space for the 8008 blending combinations, and $E^\prime$ is the matrix obtained when the number of experimental conditions n have been extracted from E. In this case study, the best $E^\prime$ selected from E was determined by the following procedure:

1. The following two steps were iterated 10,000 times:
  i. A set of n experimental conditions (i.e., n rows) was randomly selected from matrix E to form $E^\prime$.
  ii. $\det\;\left(E'^T\;E'\right)$ was calculated.
2. From the 10,000 iterations, the set of n rows that yielded the maximum value of $\det\;\left(E'^T\;E'\right)$ was identified.

Finally, $D^{\prime}$ was used to denote that these n rows were extracted from matrix D and determines how to blend the mother media in the experiment.

A visual summary of the above workflow is provided in Fig. 2e as a flowchart. In addition, the Python code for executing this workflow is available on GitHub (https://github.com/HK-bio/medium_study_repo/tree/main/paper_202504).

### Step (ⅱ): Cell culture experiment

#### Cell line and preculture

A recombinant immunoglobulin (Ig)G1 (trastuzumab)-producing Chinese hamster lung (CHL)-YN suspension cell pool was used. The CHL-YN cell line was established in our laboratory, and its parental cell line is registered as RCB5004 in the RIKEN Cell Bank, from which it is available. This IgG1-producing cell pool was established through polyethyleneimine-based transfection and puromycin-based selection, as previously detailed (Yamano-Adachi et al. 2020). All cultivations were conducted using the same working cell bank. After the stock solution was thawed, three passages of preculture were performed. During the preculture, the cell pool was cultured in EX-CELL® CD CHO Fusion medium supplemented with 6 mM l-glutamine (FUJIFILM Wako Pure Chemical, Fukuoka, Japan, Cat# 073-05391) and 5 µg/mL puromycin (Invivogen, San Diego, CA, USA, Cat# ant-pr-1) in a humidified incubator (Climo-Shaker, Kuhner, Basel, Switzerland) at 37 °C, 5% CO_2_, Φ25 mm, and 140 rpm.

#### Batch culture

Batch cultures in 24 deep well plates were conducted using the Duetz sandwich-cover system (EnzyScreen BV, Heemstede, Netherlands) and 24DW plates (EnzyScreen BV, Heemstede, Netherlands). The working volume was 2.5 mL, which consisted of 1.3 mL of EX-CELL® CD CHO Fusion medium (as the common medium) supplemented with 11.5 mM l-glutamine and 9.6 µg/mL puromycin, and 1.2 mL of mother media in total (Fig. 5a). This was to minimize the need for cell adaptation to a significantly different composition. Cells were inoculated at 5 × 10^5^ cells/mL. The cultures were performed at 37 °C, 5% CO_2_, Φ25 mm, and 300 rpm.

#### Measurement of cultured samples

Viable cell concentration (VCC) and viability were measured using a Vi-CELL BLU Cell Viability Analyzer (Beckman Coulter, Inc., Brea, CA, USA).

IgG1 concentrations in the cell supernatants were determined using a sandwich enzyme-linked immunosorbent assay, as described in a previous report (Kaneyoshi et al. 2019).

### Step (ⅲ): Regression modeling and its interpretation

As described above, the purpose of this study was to screen for the medium components X that were likely to be related to culture performance y. In both modeling and its interpretation, it is important to establish a regression model that explains the variance of y using X, as well as to ensure that the model is interpretable.

#### Modeling algorithms

An interpretable regression model is defined by its capacity to clearly indicate which variables are largely responsible for predictions via its internal weights and estimated parameters. For example, in a linear regression model, the sign and magnitude of each regression coefficient quantitatively reveal the effects of medium components on culture performance. This clarity allows researchers to more readily identify promising directions for medium optimization. However, it is known that interactions occur among medium components (Schnellbaecher et al. 2019; Pandey et al. 2024), and drastic changes may arise from component depletions or metabolic shifts. Therefore, it is necessary to consider whether some patterns might be difficult to explain using simple models. Accordingly, machine learning approaches, which can model such complex nonlinearities, have been increasingly used in recent years (Tai- et al. 2021; Hashizume et al. 2023; Hashizume and Ying 2024; Gangwar et al. 2024). However, many of these approaches operate as black boxes, resulting in cases where high predictive accuracy is accompanied by low interpretability. Thus, in models developed by machine learning, interpretability is often achieved by numerically validating the model after modeling. For example, SHapley Additive exPlanations (SHAP) values (Lundberg and Lee 2017) and permutation feature importance (PFI) (Breiman 2001) are representative of these methods, and these methods have even been applied to medium components screening (Gangwar et al. 2024). In theory, these methods can be applied to any model.

Therefore, in this study, regression modeling was carried out by considering both simple linear regression models and more complex machine learning models to account for the possibilities of interactions among medium components and the inherent complexity of cultivation. Specifically, several regression algorithms were employed corresponding to the following assumptions. First, under the assumption of drastic changes (e.g., component depletions or metabolic shifts), decision tree-based ensemble techniques—including random forest (RF) regression, gradient boosting decision tree (GBDT) regression, and extreme gradient boosting (XGB) regression—were used. Second, under the assumption of smoother multimodal behavior, algorithms that capture smooth nonlinearities—such as nonlinear Gaussian process regression (nonlinear GPR) and nonlinear support vector regression (nonlinear SVR) with a radial basis function kernel—were employed. Third, under the assumption that the observed patterns can be explained by linear relationships, linear regression techniques including partial least squares regression (PLS-R), ridge regression, least absolute shrinkage and selection operator (Lasso), elastic net regression, linear support vector regression (Linear SVR), and linear Gaussian process regression (linear GPR) were included. Multiple algorithms were chosen for each assumption to enhance interpretability and predictive robustness by identifying common trends across models.

#### Modeling procedure

In supervised learning, a smaller sample size increases the likelihood of overfitting, particularly in complex machine learning models (Ying 2019). Overfitted models tend to exhibit poor predictive accuracy on unknown data and thus reduced generalizability. Therefore, it is important to evaluate the performance of models based not only on the training set but also on unknown data (i.e., test set). In this study, model construction and evaluation were performed following a guideline for supervised-learning approaches in biological studies (Walsh et al. 2021).

The entire dataset (360 samples [120 conditions, 3 biological replicates]) was split using a 6:4 ratio into a training set and test set such that data with the same condition were consistently grouped and exclusively split into either the training set or the test set, ensuring no data leakage. Next, for hyperparameter tuning, the training set was divided using fivefold cross-validation, and the hyperparameters corresponding to the highest value (mean − standard deviation) of the predictive error (coefficient of determination; *R*^2^) on the validation set were selected. In this cross-validation, data with the same condition were also consistently grouped and exclusively assigned to either the training set or validation set. The optimized hyperparameters were used to retrain the entire training set, and then, the test set was predicted using the trained model. As performance metrics, *R*^2^ (to evaluate the extent to which the model’s predictions explain the variance of y) and the mean squared error (MSE; to identify large predictive errors) were used. Their equations are respectively as follows:

18 $$R^{2} = 1 - \frac{{\sum\limits_{i = 1}^{n} {(y_{i} - \hat{y}_{i} )^{2} } }}{{\sum\limits_{i = 1}^{n} {(y_{i} - \overline{y})^{2} } }}$$

$$\begin{gathered} \hat{y}_{i} :{\text{the predicted value for sample }}i \hfill \\ \overline{y}\,\,:{\text{the mean of }}y \hfill \\ \end{gathered}$$,

19 $${\mathrm{MSE}} = \frac{1}{n}\sum\limits_{i = 1}^{n} {(y_{i} - \hat{y}_{i} )^{2} }$$

**Fig. 3:**
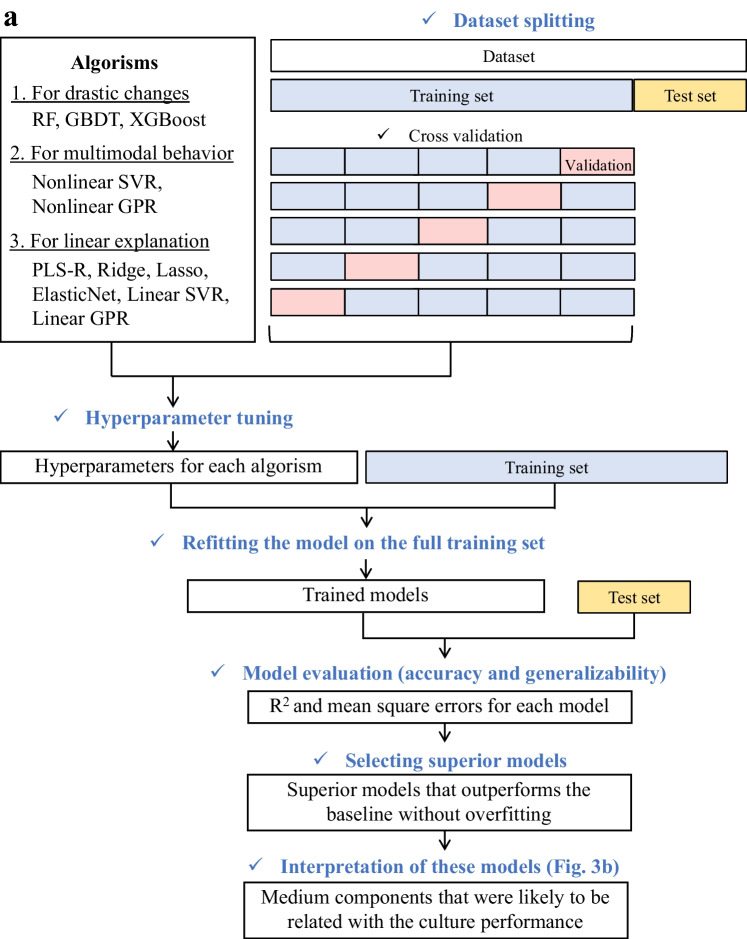

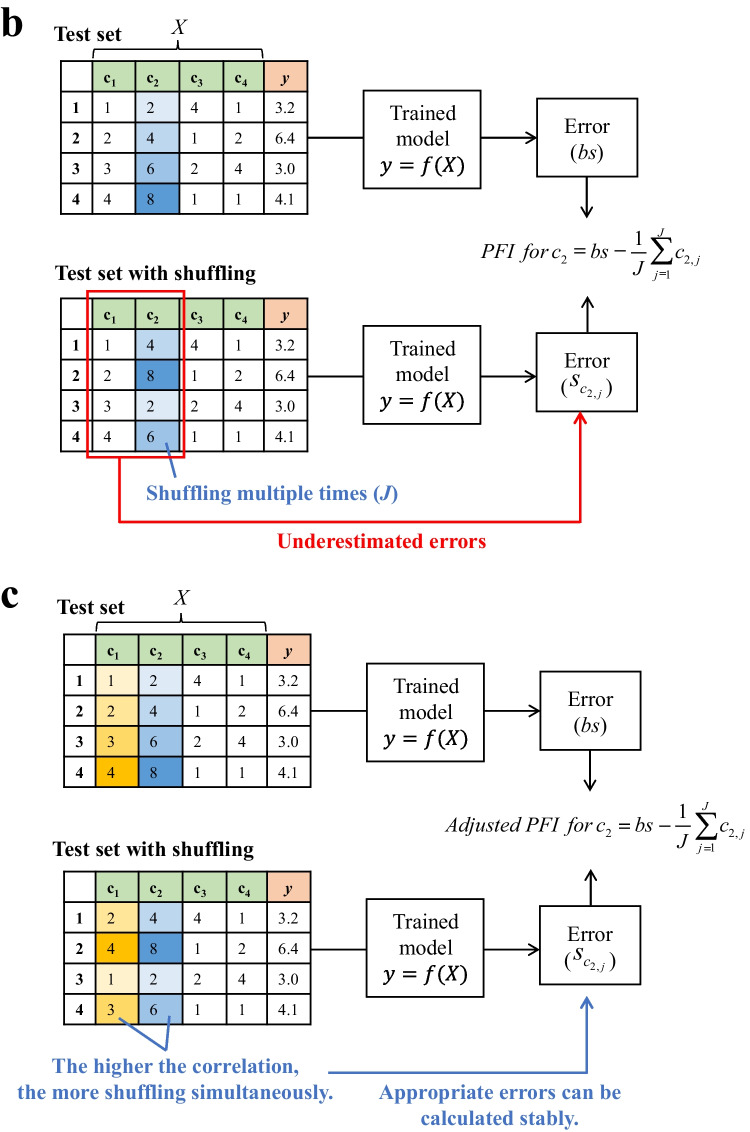
Regression modeling and the interpretation. **a** Flowchart outlining the entire process from regression modeling to interpretation. Blue text represents execution steps, whereas boxes indicate information such as data, variables, and models. **b** Graphical explanation of permutation feature importance (PFI) calculation. Here, an example of calculating the PFI for $c_{2}$ is presented. $c_{1}$ and $c_{2}$ are highly correlated, containing overlapping information for *y* = *f*(*X*). Thus, *c*_1_ compensates for the loss when $c_{2}$ is shuffled, resulting in underestimated errors. **c** Graphical explanation of the adjusted PFI calculation procedure, which considers intervariable correlation. Here, an example of calculating the adjusted PFI for $c_{2}$ is presented. The simultaneous shuffling of $c_{1}$, which highly correlates with $c_{2}$, ensures that the error for $c_{2}$ is accurately estimated without underestimation. RF, random forest regression; GBDT, gradient boosting decision tree regression; XGBoost, extreme gradient boosting regression; Nonlinear SVR, nonlinear support vector regression; Nonlinear GPR, Nonlinear Gaussian process regression; PLS-R, partial least squares regression; Ridge, ridge regression; Lasso, least absolute shrinkage and selection operator; ElasticNet, elastic net regression; Linear SVR, linear support vector regression; Linear GPR, linear Gaussian process regression

The test set was randomly split into five sets, and the MSE was computed for each set. The mean and standard error were then calculated to evaluate the significance relative to the baseline model (which predicts the mean value). The overall framework of the model construction and evaluation is shown in Fig. 3a, with details provided in Supplemental Table S2.

Finally, the models meeting the following conditions were defined as “superior models,” and consequently were used in the model interpretation step.

- Predictive accuracy: the MSE on the test data is significantly lower than that of the baseline (one-sided paired *t*-test [Holm’s correction, *α* = 0.05]).
- Generalizability: the ratios of the average *R*^2^ to MSE for the training and test datasets are within 15%.

#### Interpretation of the models

In the superior models, the PFI, which is a model-independent metric (Breiman 2001), was applied to estimate the components that are most closely related to y. The PFI is calculated as follows:

1. The *R*^2^ score (baseline score;bs) of the trained model on the test set is calculated.
2. The following is iterated multiple times (J) for all explanatory variables.
  - One explanatory variable $x_{i}$ in the test set is selected and the values are shuffled randomly.
  - The predictive score ($s_{i,j}$) is calculated with the shuffled test set using the same trained model.

(3) The PFI for $x_{i}$ is calculated using the following equation:

20 $$PFI\;for\;x_{i} = \;bs\; - \;\frac{1}{J}\sum\limits_{j = 1}^{J} {s_{i,j} } .$$

Nevertheless, in the case of media blending, the results of PFI can be misinterpreted. Although the proposed experimental design can minimize multicollinearity in the non-redundant portion among the explanatory variables as explained, multicollinearity arising from the rank deficiency of matrix A remains inevitable. Consequently, as illustrated in Fig. 2b, inherent correlations among the explanatory variables cause components that are essential for predicting y to be overlooked. Notably, the PFI of highly correlated variables tends to be underestimated relative to that of other variables (Fig. 3b). Given the objective of the initial screening of medium components, it is important to reduce the risk of overlooking key variables. Therefore, a method for the importance estimation that prioritizes sensitivity even if allowing some redundancy is necessary.

To address this issue, we adopted the method proposed by Kaneko et al. (Kaneko, 2022), which involves simultaneously shuffling groups of highly correlated variables during the calculation of PFI. This method ensures that the importances are not underestimated and are appropriately quantified (Fig. 3c). The adjusted PFI, which accounts for the correlations among variables, was calculated as follows:

1. The absolute correlation coefficient *r*_*p,q*_ between the *p*-th and *q*-th explanatory variables was calculated.
2. This $r_{p,q}$ was then transformed into $z_{p,q}$ via the Fisher z-transformation as follows:
  21 $$z_{p,q}=\frac12\,\ln(\frac{1+r_{p,q}}{1-r_{p,q}}).$$
3. With the sample size defined as m, z was assumed to follow a normal distribution with mean $z_{p,q}$ and variance $\frac{1}{m - 3}$. The lower and upper limits of the interval at significance level α were denoted by $L_{{z_{p,q} }}$ and $U_{{z_{p,q} }}$.
4. $L_{z_{p,q}}$and $U_{{z_{p,q} }}$ were converted into $L_{{r_{p,q} }}$ and $U_{{r_{p,q} }}$, respectively, which represent the lower and upper bounds of the correlation coefficient r, and calculated as follows:
  22 $$L_{r_{p,q}}=\frac{\exp(2\,L_{z_{p,q}})-1}{\exp(2\,L_{z_{p,q}})+1},\quad U_{r_{p,q}}=\frac{\exp(2\,U_{z_{p,q}})-1}{\exp(2\,U_{z_{p,q}})+1}.$$
5. If L<0<U, the correlation was considered incidental and $r^{\prime}_{p,q} = 0$ was assigned.
6. Otherwise, $r^{\prime}_{p,q} = L_{{r_{p,q} }}$ was adopted.
7. In the equation for PFI, when calculating predictive scores for the shuffled test set of the p-th explanatory variable, the following steps were carried out:
  - For each explanatory variable $q\,(q \ne p)$, the procedure below was applied to each sample in the test set:
    (i) A random number rand in [0,1] was generated.
    (ii) If $rand < r^{\prime}_{p,q}$, the corresponding sample value of explanatory variable q was shuffled as well.

### Simulation of mother media

Desirable mother media themselves were simulated using the following steps.

1. The following was iterated 100 times.
  - The following was iterated 1000 times.
    (i) Five concentration levels were randomly assigned to each component concentration value for each mother medium (e.g., 0, 1, 2, 3, and 4).
    (ii) A combination of mother media was selected in which the three penalties below were not applicable or were the smallest. A high penalty was set so that the stated condition never occurred.
      1. High penalty: variances of variables (components) and samples are zero.
      2. High penalty: the combinations of |*r*|> 0.9 between variables are included in the selected mother media.
      3. Penalty: the combinations of |*r*|> 0.7 between variables are included in the selected mother media.
  - The experimental conditions (how to blend the mother media) were determined according to the workflow as explained.
2. The mother media set that yielded the $\left(E'^T\;E'\right)$ in 100 iterations was selected as the best one.

## Results

### Experimental design of media blending for the initial screening of medium components (a case study)

**Fig. 4:**
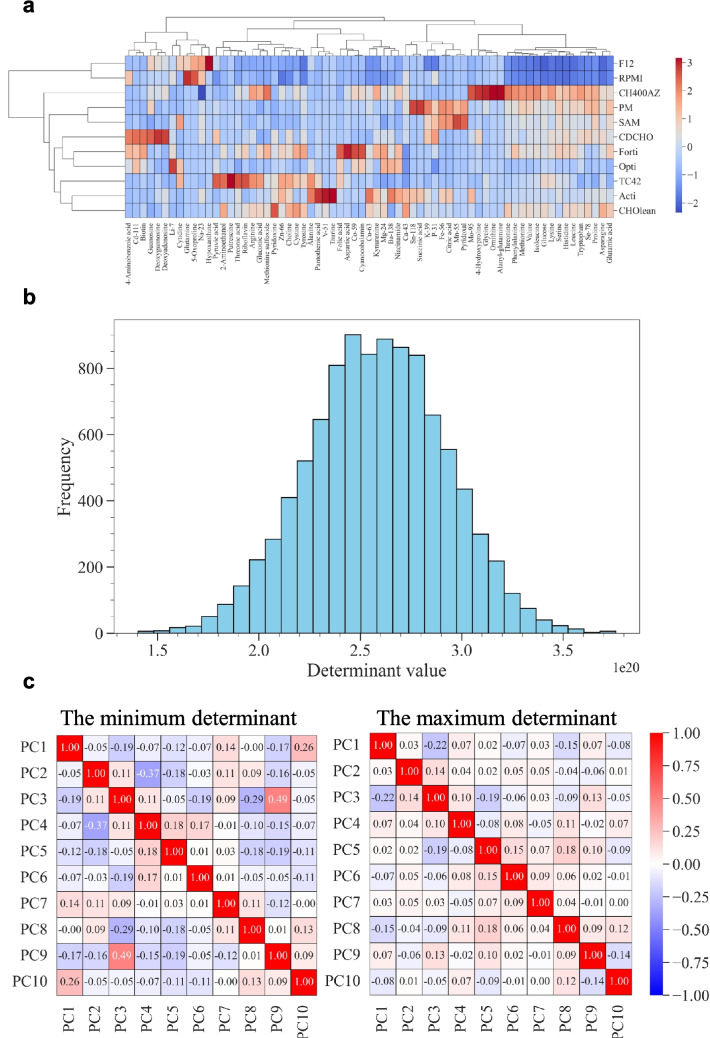
Media analysis and the selection of experimental conditions in a case study. **a** Differences in the profiles of the detected medium components among 11 types of chemically defined media used for blending. The color gradient in the heatmap indicates the relative values of each component between media: red represents high level and blue represents low level. The dendrograms above and left of the heatmap indicate the hierarchical clustering of medium components and the media, respectively, using Ward’s method with the Euclidean distance as the metric. The height at which two branches merge reflects the degree of dissimilarity between the clusters, with lower heights indicating higher similarity. F12: Ham’s F-12 Nutrient Mix; RPMI: RPMI 1640; CH400AZ: CH400AZ; PM: 4Cell® SmartCHO Media System Production Medium; SAM: 4Cell® SmartCHO Stock & Adaptation Medium; CDCHO: 1X CD CHO; Forti: CD FortiCHO™; Opti: CD OptiCHO™; TC42: TC-42 w/o GF; and Acti: HyClone™ ActiPro™. **b** Histogram of 10,000 $\det\;\left(E'^T\;E'\right)$. The experimental conditions were selected where the determinant value was the largest, located on the right-hand side of the histogram. **c** Heatmap of Pearson’s product-moment correlation coefficients of the condition with the minimum (left) and maximum (right) determinant. The lighter the red or blue, the smaller the correlation between the principal components. PC: principal component

As explained above, commercially available CDM were used as mother media to maximize simplicity and applicability for all researchers. To obtain matrix A, as defined in Eq. (1), the medium components were analyzed using LC-MS/MS and ICP-MS to determine the relative concentrations. As a result, a total of 67 components were detected and quantified. The results of hierarchical clustering based on the similarity of the medium components are shown in Fig. 4a. The CDM with the highest similarity were Ham’s F-12 Nutrient Mix and RPMI 1640. Removing one of the highly similar CDM is an option when selecting which CDM to use as the mother media. However, Ham’s F-12 Nutrient Mix (Ham 1965) and RPMI 1640 (Moore et al. 1966) were developed from different origins and have different characteristics at the component level, such as hypoxanthine and thymidine. Therefore, it was decided to use all 11 CDM as the mother media because an increase in absolute data variability could be expected.

The analytical results for CDM can be regarded as an 11 × 67 matrix A, where the number of mother media (m:11) is less than the number of medium components (d:67), as explained as the mathematical constraint in the experimental design. Therefore, PCA was applied to project matrix A onto matrix B, a low-dimensional orthogonal space that retains as much variance as possible. While it is obvious from the equations (Supplemental Note S2), it is shown that the principal components of matrix B are orthogonal, indicating no inter-correlation (i.e., multicollinearity) in Supplemental Fig. S1. Then, matrix E was calculated using Eq. (14). Multiple iterations (e.g., 10,000 iterations in this case study) were performed to randomly select the number of experimental conditions (e.g., 120 conditions in this case study) from the rows in matrix E, resulting in matrix $E^{\prime}$. The histogram of $\left(E'^T\;E'\right)$ is shown in Fig. 4b. The experimental condition with $\left(E'^T\;E'\right)$ is on the right-hand side in the histogram, and this was the condition used in the experiments. Pearson’s product-moment correlation coefficients between the principal components and variance inflation factor, which is an indicator for multicollinearity, were compared under the condition with the maximum determinant and the other conditions (the minimum and median determinant) (Table 1). As the determinant increased, both the maximum and mean absolute values of the correlation coefficients and maximum VIF tended to decrease, reaching the smallest values at the maximum determinant. Additionally, the correlation coefficients of all combinations were visualized under the conditions of providing minimum and maximum determinants (Fig. 4c). It was clearly shown that the overall uncorrelatedness improved. Thus, as expected given the mathematical explanation, the results supported that our proposed workflow enables systematic selection of the intended experimental conditions that mitigate multicollinearity among the variables.

**Table 1:** Maximum and mean absolute values of the correlation coefficients and maximum variance inflation factor (VIF) among the principal components of the medium components

	Minimum	50%	Maximum^*1^
Determinant ($\times {10}^{20}$)	1.40	2.59	3.76
Maximum |*r*|	0.491	0.327	0.219
Average |*r*|	0.118	0.092	0.075
Maximum VIF	2.11	1.41	1.20

^*^1 The condition with the maximum determinant indicates the condition to be tested

### Batch culture with the experimental conditions designed using the proposed workflow

**Fig. 5:**
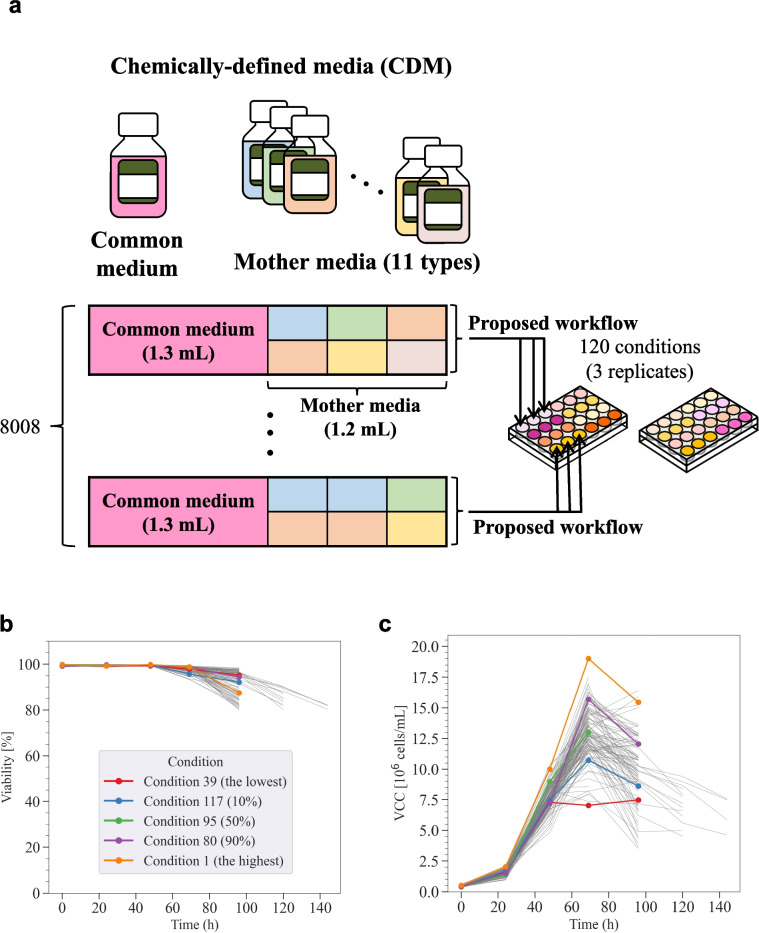

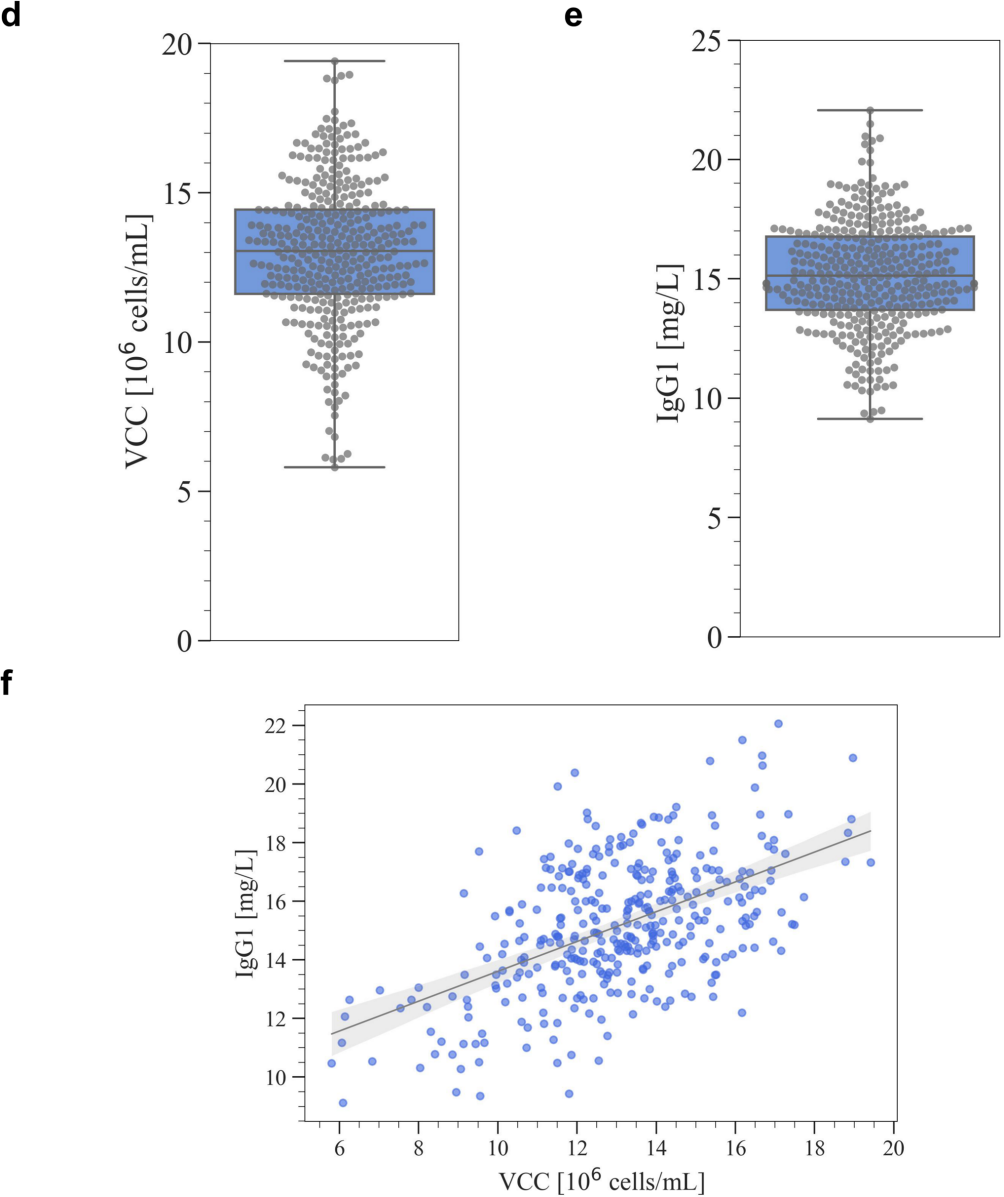
Batch culture under the selected media blending conditions. **a** Overview of the experiment in the case study. Time course of **b** viability and **c** viable cell concentrations (VCCs) under the 120 selected conditions. Batch cultures were performed with biological replicates (*n* = 3) and the results are shown within a range exceeding 80% viability. The plots indicate the mean value for 69 h (at harvest), whereas the measurement value of mixtures of replicates for values other than 69 h. The conditions shown in various colors were the lowest, lower 10%, median (50%), lower 90%, and highest VCC at harvest, respectively. Box plots of **d** VCC and **e** immunoglobulin (Ig)G1 concentration at harvest. The horizontal line within the box represents the median value, whereas the upper and lower edges of the box represent the first and third quartiles, respectively. Dots represent individual measurements, which highlight the distribution and density. **f** Scatter plot of VCC and IgG1 concentration at harvest. The shaded area represents the 99% confidence interval of the regression line

In this section, the results of the batch culture under the experimental conditions selected by the proposed workflow are presented. The screening of medium components in CHL-YN cells (Yamano-Adachi et al. 2020) was conducted as a case study. CHL-YN is a lung fibroblast-derived cell line that has similar properties to CHO cells in terms of the availability of serum-free suspension culture and a glycosylation profile. Additionally, the specific growth rate of CHL-YN cells is twice that of CHO-K1 cells. Therefore, CHL-YN is expected to be a novel host for antibody production to achieve shortened culture process development and production culture duration. However, the screening of the medium components for CHL-YN cells has not been adequately conducted. This case study was expected to assess the potential for the initial screening of components in antibody production using a new host. Batch cultures with IgG1-producing CHL-YN cells were conducted using 24 well plates under the selected 120 experimental conditions (Fig. 5a). Culture conditions for CHL-YN cells using 24 well plates were determined based on conditions reported for CHO cells (Chaturvedi et al. 2014). The changes over time in major culture parameters and extracellular metabolic components were comparable with the results in flask batch cultures (Supplemental Fig. S2). This batch culture was performed within a range exceeding 80% viability (Fig. 5b). The harvest time was set to 69 h in this case study. The stationary phase (corresponding to peak VCC) with high viability (> 95%) was expected to be recorded between 48 and 72 h in this culture condition based on preliminary studies using the EX-CELL® CD CHO Fusion medium. As expected, peak VCC were recorded at 69 h in most conditions; however, 8% in the total were recorded at 96 h (Fig. 5c). The value of VCC at 96 h in the 8% conditions was only 10% higher on average than at 69 h, which suggests that the culture was in the stationary phase from 69 to 96 h. Therefore, it was considered reasonable to set 69 h as the time for the harvest across all conditions. The VCCs at harvest were recorded from 5.8 × 10^6^ to 19.4 × 10^6^ cells/mL (Fig. 5d). The IgG1 concentrations in culture supernatants at harvest were recorded from 9.1 to 22.1 mg/L (Fig. 5e). The correlation between the VCC and IgG1 concentrations at harvest was then evaluated and the correlation coefficient was 0.54 (*p* < 0.01), which suggests that the variation in VCC was one of the reasons for the variation in IgG1 concentrations (Fig. 5f). Therefore, in this case study, an attempt was made to construct a model to explain VCC and to estimate the components that made a high contribution to the model.

### Estimation of the components related to culture performance

**Fig. 6:**
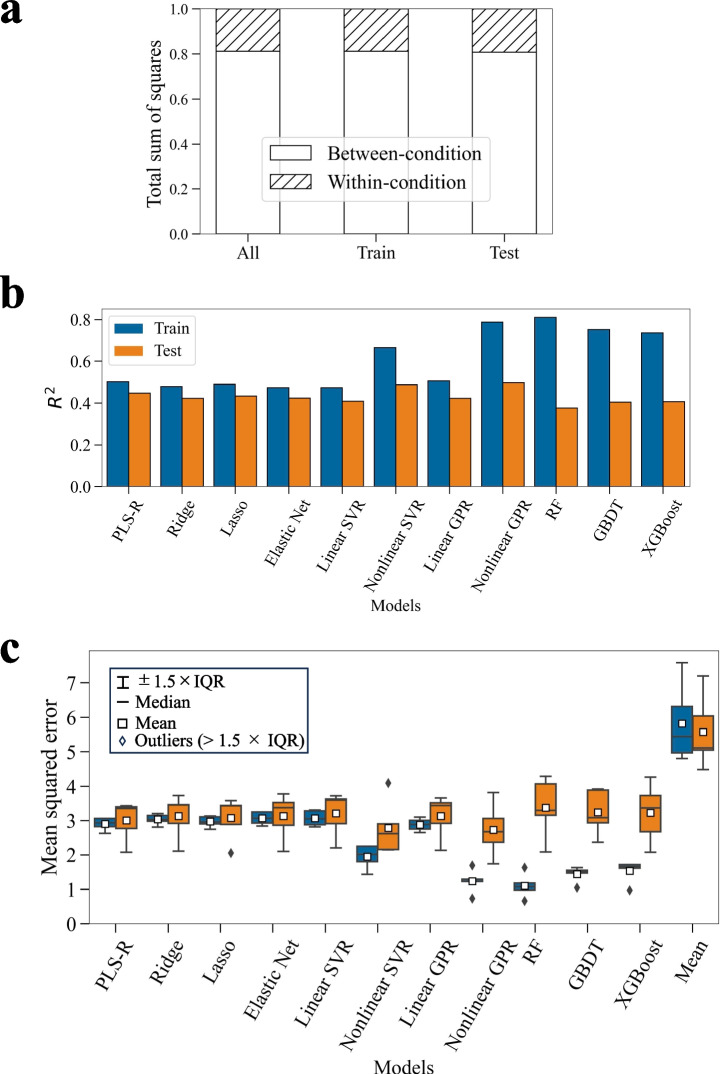

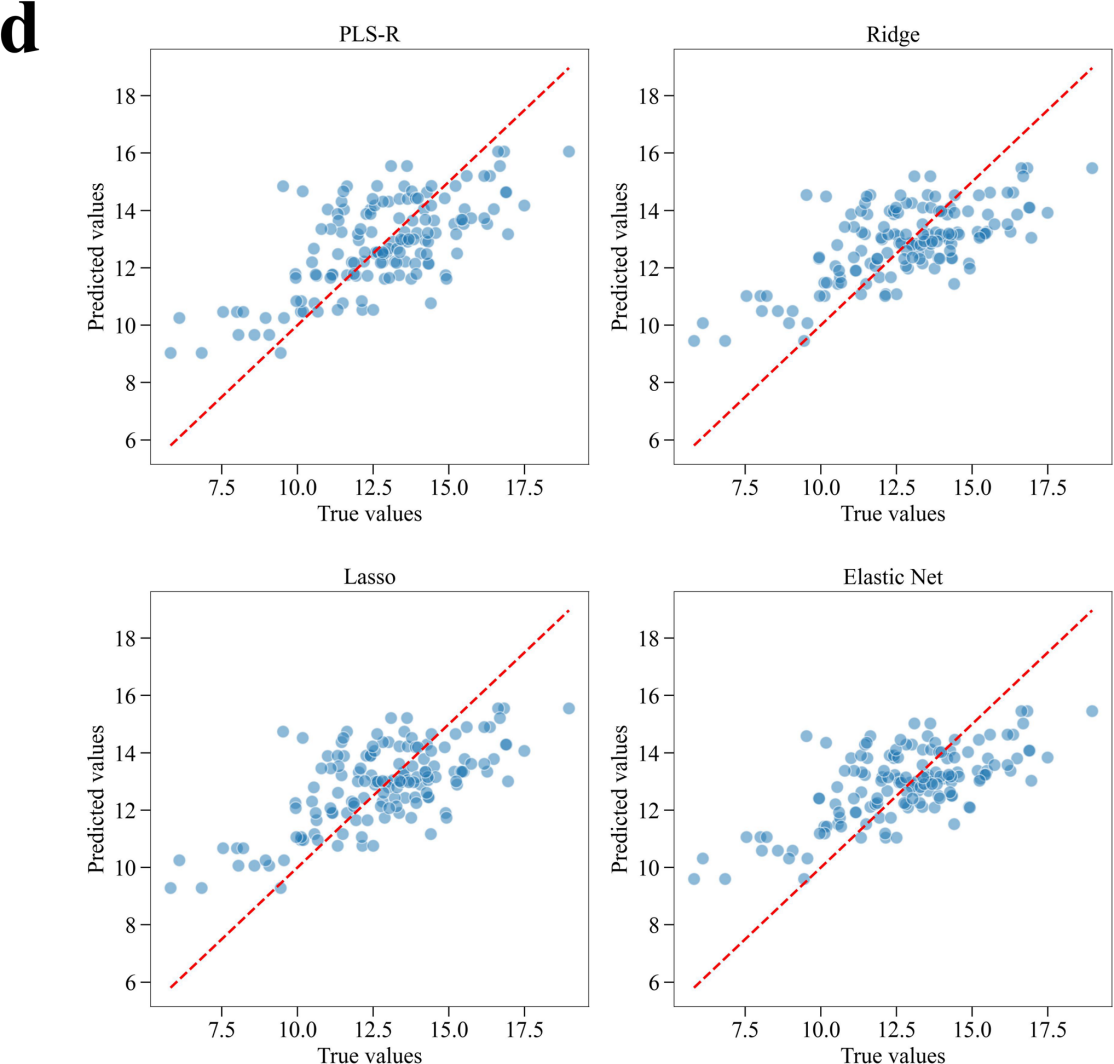
Regression modeling with VCC as the objective variable. **a** Breakdown of the sum of squares between (SSB) and within (SSW) for VCC, illustrating the ratios of SSB and SSW relative to the total sum of squares (TSS), normalized to 1 for the entire dataset, training set, and test set. **b** Coefficient of determination (*R*^2^) for the prediction of VCC at harvest by 10 different models. Blue bars indicate the prediction results for the training set and orange bars indicate the prediction results for the test set. **c** Box plots of the mean squared error for predicting VCC at harvest, comparing 10 different models with the baseline model. A one-sided paired *t*-test (Holm’s correction, *α* = 0.05) was used to compare each model with the baseline (Mean) on the test set. All models showed significantly lower MSE (*p* < 0.01). **d** Scatter plot presenting the predicted values (vertical axis) and observed (true) values (horizontal axis) in the superior models. Each point represents one sample from the test set, and the red dashed line indicates the ideal case when the predicted values equal the observed values. PLS-R, partial least squares regression; Ridge, ridge regression; Lasso, least absolute shrinkage and selection operator; Elastic Net, elastic net regression; Linear SVR, linear support vector regression; Nonlinear SVR, nonlinear support vector regression; Linear GPR, linear Gaussian process regression; Nonlinear GPR, nonlinear Gaussian process regression; RF, random forest regression; GBDT, gradient boosting decision tree regression; and XGBoost, extreme gradient boosting regression; IQR, interquartile range

An attempt was made to estimate the medium components that contributed to the variation in VCC at harvest using the dataset of batch culture with 120 experimental conditions. The variation in VCC among the conditions represented approximately 80% of the total variation (Fig. 6a), and we hence constructed regression models for this variation. The explanatory variables (X) were the theoretical relative concentrations of each component and the objective variable (y) was the VCC at harvest. A total of 10 algorithms, linear and nonlinear regression algorithms, were used under the specific assumptions as described. Model construction was performed following a guideline for supervised learning approaches in biological studies (Walsh et al. 2021) (Fig. 3a). The *R*^2^ metric directly reflects the explainability of the variation in VCC and was calculated using the entire training set and entire test set separately (Fig. 6b). Consequently, the non-linear methods achieved high *R*^2^ values on the training set; in particular, Nonlinear GPR and RF reached accuracies approaching the upper limit of *R*^2^ (0.8) as suggested by Fig. 6a. However, for these non-linear approaches, the *R*^2^ on the test set was lower than that on the training set, suggesting overfitting. In contrast, linear models exhibited comparable *R*^2^ values for both the training and test sets, indicating that they captured the fundamental structure of the entire dataset. To assess the significance of the predictive accuracy of linear models, the test set was randomly split into subsets, and the MSE and its variability were computed (Fig. 6c). Because MSE is sensitive to outliers, it was employed for evaluating large predictive errors. In this analysis, in addition to the 10 models, a baseline model that predicts the mean of VCC was also included. Similar to the results for *R*^2^, these results also showed that the nonlinear models tended to overfit, whereas this trend was not observed in the linear models. One-sided paired *t*-tests (with Holm’s correction, *α* = 0.05) were conducted between the MSE of the baseline model and those of the other models for the test set, and all models showed significantly lower MSE values (*p* < 0.01). Thus, it was suggested that the linear models were more suitable for predicting VCC than the baseline model and could capture the overall trend of the dataset without overfitting. Superior models were finally selected based on the criterion that the ratio of the average predictive error (*R*^2^ and MSE) between the training and test sets are within 15%. In this case study, the superior models were PLS-R, Ridge, Lasso, and elastic net. In Fig. 6d, scatter plots of the test set predictions using the superior models are shown, and it was observed that all models captured the overall trend of the test set.

The medium components that made a high contribution in the construction of these superior models were then estimated. We employed the adjusted PFI as a metric capable of appropriately calculating contributions even under the unavoidable partial multicollinearity.

The adjusted PFI of the four superior models were calculated. The top 25% (i.e., top 17 components) components in each model are shown in Table 2. The 12 components consistently in the top 25% among all four superior models were identified. The 12 components were asparagine, tyrosine, glucose, choline, histidine, leucine, Zn, valine, lysine, 5-oxoproline, Cu, and methionine. Most of these components were regarded as reasonable choices, based on previous findings with CHO cells and the characteristics of CHL-YN cells (a detailed interpretation is given in the Discussion part). Additionally, PLS-R was among the superior models, which facilitates the use of the variable importance in projection (VIP) score, which is a metric capable of appropriately computing variable contributions even when multicollinearity exists (Wold et al. 2001). When the 12 components were compared with the result based on the VIP score, 10 of the components were the same (Supplemental Table S3). This suggested that the observed results are largely consistent with the historically validated metric. Consequently, the entire process (from experimental design to modeling and its interpretation) of screening medium components using media blending was successfully implemented as a systematic workflow that eliminated the need for human intervention.

**Table 2:**
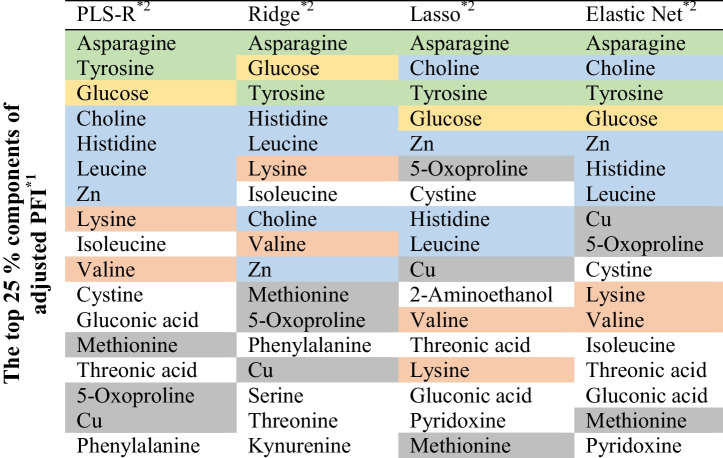
Top 25% components of adjusted permutation feature importance (PFI) in superior models

^*^1 The components identified as common among the superior models are indicated as follows: green: common in the top 5%, yellow: common in the top 10%, blue: common in the top 15%, orange: common in the top 20%, and gray: common in the top 25%

^*^2 PLS-R: partial least squares regression; Ridge: ridge regression; Lasso: least absolute shrinkage and selection operator; Elastic Net: elastic net regression

### Simulation of the mother media

We have shown that the proposed workflow can be used to systematically select components to be optimized throughout media blending. By contrast, the availability of a commercially available mother media set designed primarily for media blending with disclosed compositions would be more desirable to further enhance the interpretability of screening results. Now that the mathematical process has been established, we can conduct simulations based on a quantitative index. At the end of the study, we simulated the appropriate composition of mother media for the purpose of evaluating the benefits of developing such a mother media set and raising awareness of the development of the foundation for media blending.

**Fig. 7:**
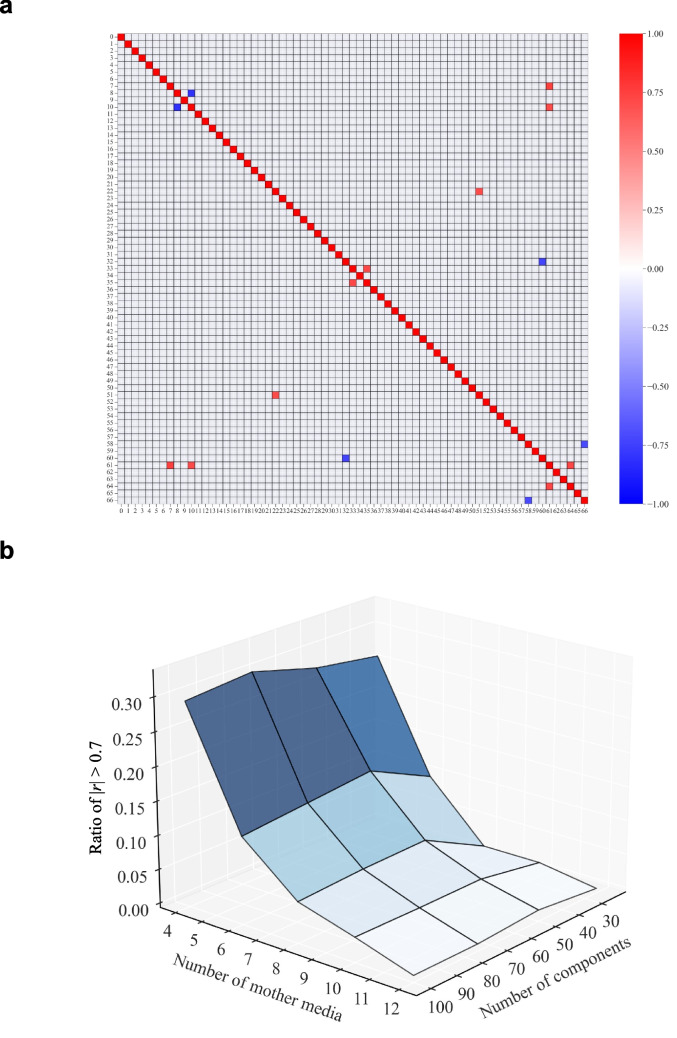
Simulation of mother media. **a** Heatmap of Pearson’s product-moment correlation coefficients (*r*) between medium components in 120 experimental conditions using simulated mother media. The simulation setup conditions are the same as those used in the case study (number of mother media: 11, number of media blending: 6, number of media components: 67). Only combinations with |*r*|> 0.7, which indicates high correlation, are shown. Red represents *r* > 0.7 and blue represents *r* < 0.7. **b** Ratios of combinations with |*r*|> 0.7 (*z*-axis) between medium components in 120 experimental conditions in each parameter. The parameters are the number of components and mother media. The number of components was set to 25, 50, 75, and 100. The number of mother media was set to 4, 6, 8, 10, and 12

First, we simulated and defined a mother media set in the same condition of the case study described in the “Breakdown of each matrix” section (number of mother media: 11, number of medium components: 67, number of experimental conditions: 120). As a result, the ratio of combinations for which |*r*|> 0.7 among the medium components in 11 different mother media was 0.05% in the simulation. This was sufficiently low compared with the 12% of combinations in this case study using CDM as mother media. Furthermore, blending these simulated mother media with our proposed workflow resulted in an average |*r*| of 0.26 among the medium components under 120 experimental conditions, and the ratio of combinations with |*r*|> 0.7 was 0.36% (Fig. 7a). For 67 medium components, the number of combinations between two components was 2211. Thus, 0.36% corresponds to approximately only eight combinations. This indicates that the number of highly correlated combinations was less than one per component, on average. Researchers may consider it sufficiently easy to interpret the importance of each variable in this scenario. These results suggest that the development of a dedicated mother media set for media blending improved the effectiveness of interpretation.

Next, we evaluated the number of mother media sufficient for the number of medium components to be screened. We simulated the number of components to be screened varied at 25, 50, 75, and 100, and the number of mother media varied at 4, 6, 8, 10, and 12. In this simulation, we generated 120 experimental conditions by media blending with our proposed workflow. Figure 7 b shows the ratios of combinations with |*r*|> 0.7 for each pairing of the number of components and number of mother media. Interestingly, at the same number of mother media, the number of components to be screened had no effect on the ratio of combinations |*r*|> 0.7. By contrast, the number of mother media seemed to dominate the effect on highly correlated combinations. Even when the number of components to be screened was 100, the number of highly correlated combinations was 16 when the number of mother media was 12. Thus, we considered that 12 mother media would be sufficient in most cases. However, because increasing the number of mother media is critical to cost and work efficiency, it is better to determine the number of mother media based on the number of components to be screened.

Although commercially available CDM as mother media and our proposed method will allow all researchers to begin screening medium components soon, the development of a commercially available mother media set designed for media blending would further enhance the usefulness of this approach, as shown in this section.

## Discussion

In this study, we proposed a workflow for the initial screening of medium components through media blending. This workflow consists of three steps: (ⅰ) experimental design, (ⅱ) cell culture experiment, and (ⅲ) regression modeling and its interpretation. To establish a systematic experimental design method, we mathematically redefined media blending for the first time and formulated the problem to be solved in the experimental design step. Media blending can be treated as a matrix calculation, and to efficiently execute the screening of medium components, we proposed the fundamental strategy of maximizing the volume of parallelepiped, as illustrated in Fig. 2c. This approach corresponds to D-optimal design in the context of DoE, and it was deemed a reasonable means for selecting appropriate experimental conditions, even with observational data. By contrast, we clarified a constraint (multicollinearity), which is inherent to media blending because the number of mother media is smaller than the number of medium components. This constraint poses challenges to systematic experimental design with the standard use of D-optimal design. Consequently, we explored a dimensionality reduction method aimed at reducing the matrix to a vector space free of multicollinearity while retaining the variance in the mother media. Finally, the dimensionality reduction method is equivalent to PCA (Fig. 2d). Using PCA for dimensionality reduction, we were able to execute the experimental design under the specific mathematical constraint of media blending. The overall workflow for the experimental design is summarized in the flowchart in Fig. 2e. When constructing and interpreting the models, we built models using both linear and nonlinear approaches under the assumption that the medium components exhibit complex behavior. We then comprehensively evaluated their predictive performance and generalizability to define the superior models. Given that the set of superior models might include multiple types, we applied a PFI-based method that can estimate variable contributions regardless of the model type. However, multicollinearity remained a challenge at this stage, as conventional PFI tends to underestimate the contributions of highly correlated variables. To address this issue, we introduced an adjusted PFI that groups highly correlated variables for evaluation, thereby enabling a more accurate estimation of their contributions (Fig. 3c). Finally, we proposed a systematic and feasible workflow that could handle the entire process from experimental design to data analysis based on quantitative indices.

We applied the workflow to medium components screening for IgG1-producing CHL-YN cells. This enabled the identification of the 12 components set that could be related to the variation of the VCC from the 67 components detected in the CDM. The next stage of optimization allowed us to determine the optimal compositions of these components in a basal or feed medium suitable for CHL-YN cells. The 12 components were asparagine, tyrosine, glucose, choline, histidine, leucine, Zn, valine, lysine, 5-oxoproline, Cu, and methionine. Notably, asparagine, tyrosine, and glucose were common in the top 10% among all models. Glucose is clearly an important carbon source that accounts for 65% of the influx carbon reported in a metabolic flux analysis study using CHO-K1 cells (Nicolae et al. 2014). We previously reported that CHL-YN cells also require glucose in glucose-controlled fed-batch cultures (Sukwattananipaat et al. 2024). Therefore, we assumed that glucose was one of the top factors that could be related to VCC in CHL-YN batch culture. Asparagine depletion has been shown to severely limit cell growth, even in the presence of aspartic acid in GS-CHO (Duarte et al. 2014). Asparagine is converted to aspartic acid and used to supply intermediates in the tricarboxylic acid (TCA) cycle and is also considered important in the supply of arginine to maintain the ornithine synthesis pathway in CHL-YN cells (Sukwattananipaat et al. 2024). The ability to produce ornithine is a metabolic feature of CHL-YN cells that is absent in CHO-K1 cells, and this is supported by the mRNA expression of arginase-2 (Sukwattananipaat et al. 2024). Ornithine is used as a precursor to reactions that produce polyamines and glutamic acid. In a previous study, researchers indicated that polyamines enhance cell growth in CHO cells (Capella Roca et al. 2019). In our previous analysis of putrescine, one of the polyamines, we did not detect it in CHO-K1 cells, whereas we did detect it in CHL-YN cells, which suggests that polyamines are produced in CHL-YN cells. Glutamic acid is used to produce not only α-ketoglutaric acid, an intermediate in the TCA cycle, but also glutamine in CHL-YN cells. CHL-YN cells can produce glutamine because the expression level of glutamine synthase is higher than that in CHO cells (Yamano-Adachi et al. 2020). Glutamine is produced by glutamine synthase using glutamic acid and ammonia as substrates. Given that excessive accumulation of ammonia is widely known to induce cytotoxicity in CHO cells (Synoground et al. 2021), we expect glutamine synthesis ability in CHL-YN cells to be advantageous for cell growth. From the above, it is reasonable that asparagine was consistently of high importance in all superior models in this study because it is involved in several important metabolic reactions in CHL-YN cells. Tyrosine has been shown to suppress endoplasmic reticulum (ER) stress and apoptosis in CHO cells, accompanied by the synthesis of coenzyme Q10 (Shibafuji et al. 2023). Moreover, the depletion of tyrosine during culture is known to result in decreased viable cell concentrations (Zhang et al. 2020). Considering that tyrosine is sharply consumed in CHL-YN cells (Sukwattananipaat et al. 2024), the identification of tyrosine is also deemed a reasonable result.

Clearly, it is worth noting that this workflow is also applicable to commercially available CDM, as demonstrated in the case study. The use of commercially available CDM designed for the intended purpose would enable researchers to evaluate the appropriate concentration ranges without requiring extensive prior knowledge. When researchers screen components for new hosts, as conducted in this study, the use of mother media with considerably different profiles, such as Ham’s F-12 Nutrient Mix or RPMI 1640, would be effective to evaluate a wide range of concentrations. In this case, the common medium used in the pre-culture should be included to minimize the need for cell adaptation to a significantly different composition. Therefore, two types of designs should be considered: one where a fixed amount of the common medium is used for evaluation and another where the entire working volume is subject to blending mother media without the common medium. Scripts for both types of experimental design are available on GitHub (https://github.com/HK-bio/medium_study_repo/tree/main/paper_202504).

In this study, we were the first to mathematically explain the constraints of media blending. Although we proposed a workflow that works under these constraints, it is still necessary to acknowledge that the results obtained from this workflow need to be interpreted carefully. In the modeling and its interpretation, we applied adjusted PFI to multiple models, which allowed for the appropriate calculation of importance, even in the presence of multicollinearity (Fig. 3c). The approach successfully narrowed down the number of medium components of interest. We expect the reduction in the number of variables to be handled to make subsequent optimization work feasible. However, we recognize that it is limited only to the narrowing down of the component “set.” In the presence of multicollinearity, it is not possible to assess the effect of individual medium components and the interactions. Thus, after narrowing down the components to a manageable number through initial screening with media blending, we should acknowledge that it is necessary to verify the effects of individual components and optimize their concentrations using optimization methods (e.g., factorial design or Bayesian optimization). It is important to note that if multicollinearity can be eliminated, the effects and interactions of individual components can be assessed in a single step, but this diminishes the advantages of the simplicity of media blending. As explained thus far, mathematically eliminating multicollinearity requires that the number of mother media is at least equal to the number of components. The primary advantage of media blending is its ability to easily screen multiple components; hence, preparing more mother media than the number of components is impractical and diminishes the convenience of media blending. From this perspective, the workflow presented in this study can serve as a general guideline for conducting media blending.

Regarding the key component set identified by a data-driven approach, genome-scale models (GEMs) can be useful for evaluating the effects and interpretability of individual components. GEMs computationally describe a whole set of stoichiometry-based, mass-balanced metabolic reactions using gene-protein reactions, and their application to media design has been advancing (Park et al. 2024a). When a linear relationship between component concentration and specific consumption rate can be assumed, it is possible to simulate the effect of the medium composition on culture performance by arbitrarily changing the constraint values (in this case, the specific consumption rates of components) in flux balance analysis (FBA). For example, Pang et al. (2024) simulated the culture performance by varying the asparagine/aspartic acid ratio in enzyme capacity-constrained FBA (ecFBA). Additionally, several studies combining data-driven approaches and genome-scale models have been reported. For example, Hong et al. (2022) identified key metabolites that distinguish differences between culture conditions, based on VIP scores derived from partial least squares discriminant analysis (PLS-DA), and then detected enriched pathways (specifically, TCA cycle) by metabolite set enrichment analysis. By estimating fluxes using ecFBA with a GEM, specific metabolic bottlenecks in the TCA cycle were identified. Finally, simulation results suggested that increasing the specific consumption rate of coenzyme Q10 would resolve the bottleneck, and this was verified by supplementing coenzyme Q10. This approach has been applied not only to basal media (Yeo et al. 2022; Hong et al. 2022) but also to feed media (Park et al. 2023, 2024b). Such a combination of data-driven approaches and GEMs enables both structural understanding of experimental data and interpretability, thereby offering a promising direction for further improvements in medium design efficiency. Since the construction of iCHO1766 (Hefzi et al. 2016), GEMs of CHO cells have been improved by increasing the number of reactions and developing contextualized models specific to cell lines. CHL-YN cells were also derived from a Chinese hamster but have metabolic characteristics that differ from those of CHO cells (Yamano-Adachi et al. 2020; Sukwattananipaat et al. 2024). We are currently developing a GEM specific to CHL-YN cells, and it is expected to be applied.

We recognize that the present study has been confined to CHL-YN cells, and we consider this a limitation in terms of demonstrating the full range of applications. Nevertheless, the underlying formalized methodology is designed to be broadly applicable, and we expect for it to be effectively adapted to other cell types in future studies. In addition to the scope of application with other cell types, it is important to consider the limitations imposed by the sample size in bioprocessing applications. In this study, we employed a small-scale culture with a total culture volume of 2.5 mL, which allowed us to obtain data from 360 samples. However, in industrial bioprocessing applications using bioreactors, it is often only feasible to test a limited number of conditions per culture. In supervised learning, a smaller sample size increases the likelihood of overfitting, particularly in complex machine-learning models (Ying 2019). Therefore, when deploying such nonlinear models, it is essential to rigorously assess their generalizability. Recent studies have reported that transfer learning and meta-learning can be successfully applied to bioprocessing, facilitating the development of effective models from limited samples (Helleckes et al. 2024). Furthermore, hybrid modeling that combines data-driven modeling and kinetic modeling is also expected to be a method for improving generalizability (Narayanan et al. 2023). Kinetic models are expressed as differential–algebraic equations and formalize knowledge based on physical principles, thereby providing a reasonable explanation of the general behavior of the cell culture system. It is possible to improve both predictive accuracy and generalizability by expressing the general behavior using kinetic models and fine-tuning the complex nonlinearities specific to cell lines or culture conditions using data-driven models. For example, Okamura et al. (2022) constructed a kinetic model using mass balance equations and Monod equations, and then used a data-driven model to update predictions for metabolites such as lactate which show non-linear and drastic changes and fed these corrections back to adjust the model parameters. As a result, it was suggested that hybrid modeling can improve flexibility in modeling. Therefore, it is expected that the use of new machine learning techniques such as transfer learning and the integration with kinetic modeling will enable the construction of generalizable models with the benefit of non-linear explainability of data-driven approaches.

We await the availability of mother media dedicated to media blending from suppliers. This means that, in addition to the simplicity of media blending, interpretability is maximized through compositional disclosure and the minimization of correlations between components. This is precisely what is now possible because the mathematical understanding and proposed workflow enabled the design of mother media through simulation, as shown in this study. We showed that the development of a dedicated mother media set further improves the interpretability of the results based on correlations. In the culture experiment case study, 12% of all combinations had |*r*|> 0.7, whereas simulations under the same conditions showed only 0.05% of all combinations among medium components. Additionally, it is important to focus on increasing the number of mother media rather than decreasing the number of components to be screened to reduce correlations among components as much as possible. Clearly, increasing the number of mother media increases complexity, which leads to decreased work efficiency and increased costs; hence, it is important to define appropriate criteria. For example, a criterion such as “no more than one highly correlated combination per component” could be considered.

In terms of practicality, the number of culture experimental conditions in one operation varies depending on each experiment. D-optimal design allows researchers to determine the number of experimental conditions to be cultured optionally. In the experiment in this study, we performed pipetting manually for blending media, but it can be automated using a liquid handling system. We are currently working on the automation of pipette manipulation. We plan to provide code for automation that can be incorporated into the workflow presented in this study.

To summarize, we outlined the challenges to progress in media blending. This meant reassessing media blending from a mathematical perspective. Finally, we presented a systematic workflow for screening medium components throughout media blending. The workflow can be applied to any case, including the case using commercially available CDM. Additionally, the mathematical process throughout the entire step made it possible to design appropriate mother media sets backward. The workflow and simulation scripts presented in this study are publicly accessible. We look forward to the results of this study leading to further media blending developments in the future.

## Supplementary information

Below is the link to the electronic supplementary material.

ESM1 (PDF 262 KB)

ESM2 (PDF 966 KB)

## Data Availability

The code for experimental design in media blending described in Fig. 2e is publicly available from the ‘Workflow_blending_media’ folder in the GitHub repository (https://github.com/HK-bio/medium_study_repo/tree/main/paper_202504). The code for regression modeling and evaluation described in Fig. 3a are publicly available from the ‘Modeling’ folder in the same repository. The code for the simulation of mother media set is available from the ‘Simulation_of_mother_media’ folder in the same repository. The raw data of media analysis in Fig. 2a is not publicly available due to the risk of reverse engineering causing a loss of profit for the companies developing cell culture media.
