# Supplementary material for: Redefining media blending mathematically: a systematic approach for screening of medium components
Source: Appl Microbiol Biotechnol. 2025 Dec 8;110:1. doi: 10.1007/s00253-025-13594-z (PMC12764549; doi:10.1007/s00253-025-13594-z)
Supplement: Supplementary file 1 [file 253_2025_13594_MOESM1_ESM.pdf]

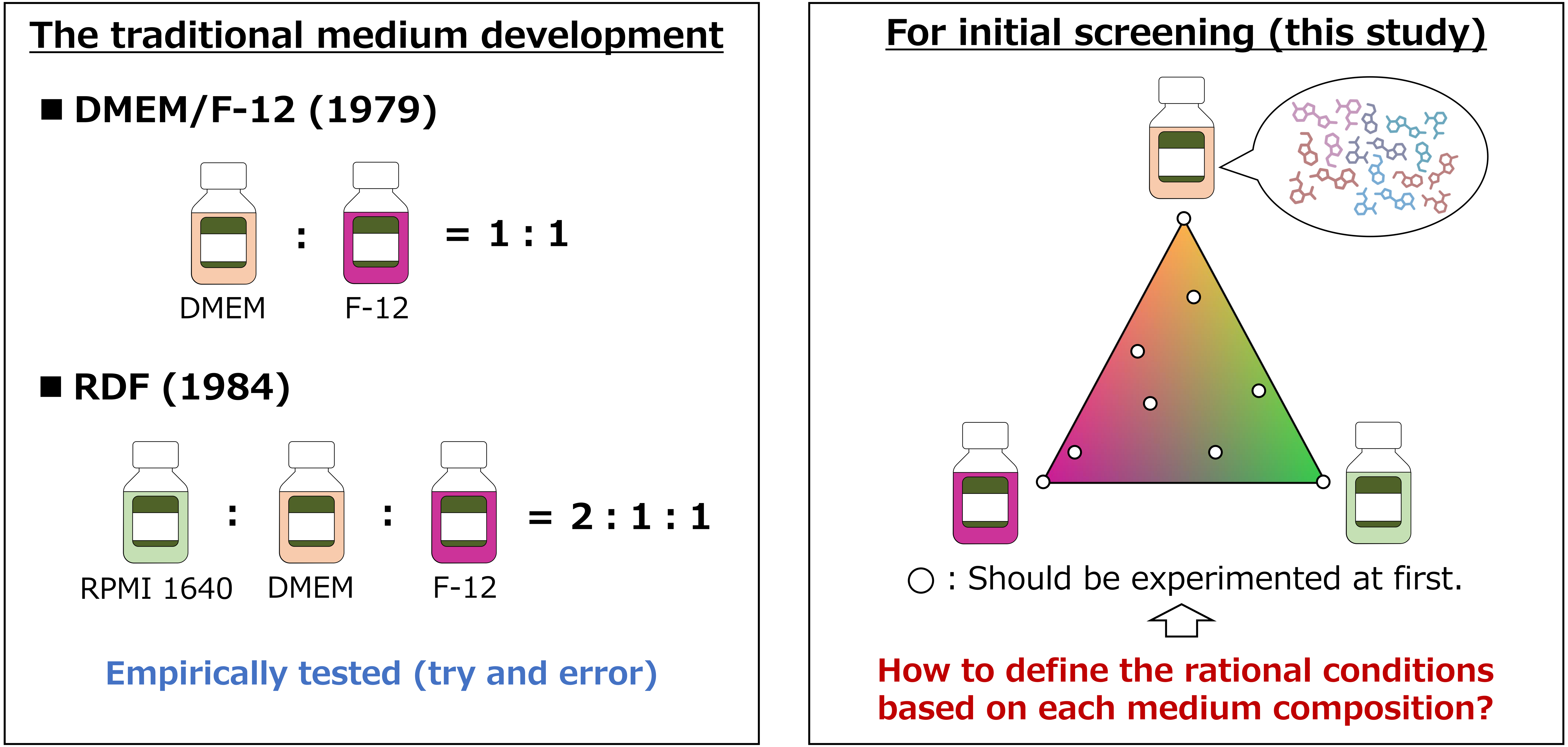

The traditional medium development
■ DMEM/F-12 (1979)
:
= 1 : 1
DMEM
F-12
■ RDF (1984)
:
:
= 2 : 1 : 1
RPMI 1640
DMEM
F-12
Empirically tested (try and error)
For initial screening (this study)
○ : Should be experimented at first.
How to define the rational conditions
based on each medium composition?
