# Supplementary material for: Redefining media blending mathematically: a systematic approach for screening of medium components
Source: Appl Microbiol Biotechnol. 2025 Dec 8;110(1):1. doi: 10.1007/s00253-025-13594-z (PMC12764549; doi:10.1007/s00253-025-13594-z)
Supplement: Supplementary file 2 — (PDF 966 KB) [file 253_2025_13594_MOESM2_ESM.pdf]

Redefining media blending mathematically: a systematic approach for screening of medium components

Hirotaka Kuroda[a, b, c], Kazuya Sorada[a, b, c], Noriko Yamano-Adachi[a, d, e*] and Takeshi Omasa[a, d, e]

[a]Graduate School of Engineering, Osaka University, 2-1 Yamadaoka, Suita, Osaka 565-0871, JAPAN; [b]Shimadzu Corp., 1 Nishinokyo Kuwabara, Nakagyo-ku, Kyoto 604-8511, JAPAN; [c]Shimadzu Analytical Innovation Research Laboratory, Osaka University, 2-1 Yamadaoka, Suita, Osaka 565-0871, JAPAN; [d]Institute for Open and Transdisciplinary Research Initiatives, Osaka University, 2-1 Yamadaoka, Suita, Osaka 565-0871, JAPAN; [e]Manufacturing Technology Association of Biologics (MAB), 7-1-49 Minatojima-Minamimachi, Chuo, Kobe, Hyogo 650-0047, JAPAN

* Noriko Yamano-Adachi, Ph.D.: yamanori@bio.eng.osaka-u.ac.jp

Table of contents

Supplemental Notes 2

Supplemental Note S1 Explanation of why the determinant is zero when $E'^T E'$ is rank deficient. 2

Supplemental Note S2 The dimensionality reduction that achieves free of multicollinearity and minimizing the loss of the variance. 4

Supplemental Tables 9

Supplemental Table S1 Analytical conditions for the measurement of metal ions using ICPMS-2030 9

Supplemental Table S2 Details of model construction according to a guideline for supervised learning approaches in biological studies. 10

Supplemental Table S3 Top 12 components of variable importance in projection (VIP) score in PLS-R model and adjusted permutation feature importance (PFI) 12

Supplemental Figures 13

Supplemental Fig. S1 Heatmap of the pearson's product-moment correlation coefficients between principal components of the medium components in 11 types of chemically-defined media. 13

Supplemental Fig. S2 Comparison of the culture results of CHL-YN between 24 well plate and flask. Batch cultures were carried out with biological replicates (*n* = 4). 14

Supplemental Notes

Supplemental Note S1 Explanation of why the determinant is zero when $E'^{T}E'$ is rank deficient.

As explained in equation (4) of the main text, E' is defined as follows:

$$E' = E_{I,:}, \quad |I| = n, \quad E' \in \mathbb{R}^{n\times d}$$

Since $E'^{T}E' \in \mathbb{R}^{d\times d}$ is a symmetric matrix, it can be eigen-decomposed as follows:

$$E'^{T}E' \in \mathbb{R}^{d\times d} = Q\Lambda Q^{-1}, \quad \Lambda = \mathrm{diag}(\lambda_1, \lambda_2, \ldots, \lambda_d), \quad Q^{T}Q = I, \quad E'^{T}E'Q = Q\Lambda.$$

Therefore,

$$\begin{aligned}\det\left(E'^{T}E'\right) &= \det\left(Q\Lambda Q^{-1}\right)\\ &= \det(Q)\det(\Lambda)\det(Q^{-1})\\ &= \det(QQ^{-1})\det(\Lambda)\\ &= \det(I)\det(\Lambda)\\ &= \det(\Lambda)\end{aligned}$$

By the definition of the determinant,

$$\det(\Lambda) = \sum_{\sigma\in S_n} \mathrm{sgn}(\sigma)\prod_{i=1}^{n}\Lambda_{i,\sigma(i)}$$

Since all non-diagonal entries of Λ are zero, this equation can be expressed accordingly.

$$\prod_{i=1}^{n}\Lambda_{i,\sigma(i)} = \begin{cases}\prod_{i=1}^{n}\lambda_i, & \text{if } \sigma(i) = i \quad \forall i,\\ 0, & \text{otherwise.}\end{cases}$$

Therefore,

$$\det\left(E'^{T}E'\right) = \det(\Lambda) = \prod_{i=1}^{d}\lambda_i.$$

From equation (11) in the main text, we know that $\mathrm{rank}\left(E'^{T}E'\right) \le m < d$. By the Rank-Nullity Theorem,

$$\mathrm{rank}\left(E'^{T}E'\right) + \mathrm{nullity}\left(E'^{T}E'\right) = d.$$

That is,

$$\text{nullity}\left(E'^{T}E'\right) \geq \begin{cases} d - m \geq 1, & \text{if } m < n, \\ d - n \geq 1, & \text{if } n < m. \end{cases}$$

Since the number of eigenvalues $\lambda = 0$ in the matrix Λ equals $\text{nullity}\left(E'^{T}E'\right)$, there is at least one zero eigenvalue. Therefore,

$$\prod_{i=1}^{d} \lambda_i = 0$$

$$\therefore \quad \det\left(E'^{T}E'\right) = 0.$$

Based on the above, if rank deficiency occurs in $E'^{T}E'$, then its determinant $\det\left(E'^{T}E'\right)$ is always zero.

Supplemental Note S2 The dimensionality reduction that achieves free of multicollinearity and minimizing the loss of the variance.

We pursued dimensionality reduction that achieves two objectives: reducing the data to a vector space free of multicollinearity and minimizing the loss of the original data's variance.

We begin by standardizing the variables in the matrix A to form the matrix $A' \in \mathbb{R}^{m\times d}$, and denote the i-th row vector of A' by $\mathbf{a}_i \in \mathbb{R}^d$. When $\mathbf{a}_i$ is orthogonally projected onto the basis vector $\mathbf{v}_1 \in \mathbb{R}^d$ (with $\|\mathbf{v}_1\| = 1$, i.e., $\mathbf{v}_1^\top \mathbf{v}_1 = 1$), denote the projected value by z_i;

$$z_i = \mathbf{a}_i \mathbf{v}_1 \tag{2-1}$$

We denote by $\mathrm{Var}(\mathbf{z})$ the variance of the projected data $\mathbf{z} \in \mathbb{R}^m$. Then,

$$\begin{aligned}\mathrm{Var}(\mathbf{z}) &= \frac{1}{m}\sum_{i=1}^{m} z_i^2 \\ &= \frac{1}{m}\sum_{i=1}^{m} (\mathbf{a}_i \mathbf{v}_1)^2.\end{aligned} \tag{2-2}$$

Since $\mathbf{a}_i \mathbf{v}_1$ is a scalar, it follows that

$$\begin{aligned}(\mathbf{a}_i \mathbf{v}_1)^2 &= (\mathbf{a}_i \mathbf{v}_1)(\mathbf{a}_i \mathbf{v}_1) \\ &= (\mathbf{v}_1^\top \mathbf{a}_i^\top)(\mathbf{a}_i \mathbf{v}_1).\end{aligned} \tag{2-3}$$

Thus,

$$\begin{aligned}\mathrm{Var}(\mathbf{z}) &= \frac{1}{m}\sum_{i=1}^{m} (\mathbf{v}_1^\top \mathbf{a}_i^\top)(\mathbf{a}_i \mathbf{v}_1) \\ &= \mathbf{v}_1^\top \left(\frac{1}{m}\sum_{i=1}^{m} \mathbf{a}_i^\top \mathbf{a}_i \right) \mathbf{v}_1.\end{aligned} \tag{2-4}$$

The matrix $\frac{1}{m}\sum_{i=1}^{m} \mathbf{a}_i^\top \mathbf{a}_i$ is the covariance matrix of A', which we denote by $\mathbf{\Sigma} = \frac{1}{m}\sum_{i=1}^{m} \mathbf{a}_i^\top \mathbf{a}_i$. Therefore,

$$\mathrm{Var}(\mathbf{z}) = \mathbf{v}_1^\top \mathbf{\Sigma} \mathbf{v}_1. \tag{2-5}$$

Finding the basis vector $\mathbf{v}_1$ that maximizes $\mathrm{Var}(\mathbf{z})$ is equivalent to solving $\max \mathbf{v}_1^\top \boldsymbol{\Sigma} \mathbf{v}_1$ subject to the constraint:

$$g(\mathbf{v}_1) = \mathbf{v}_1^\top \mathbf{v}_1 - 1 = 0. \qquad (2\text{-}6)$$

We denote the Lagrange multiplier by λ, and define the Lagrangian function $\mathcal{L}(\mathbf{v}_1, \lambda)$ as

$$\mathcal{L}(\mathbf{v}_1, \lambda) = \mathbf{v}_1^\top \boldsymbol{\Sigma} \mathbf{v}_1 + \lambda(\mathbf{v}_1^\top \mathbf{v}_1 - 1) \qquad (2\text{-}7)$$

By applying the method of Lagrange multipliers, we set $\frac{\partial \mathcal{L}}{\partial \mathbf{v}_1} = 0$ and $\frac{\partial \mathcal{L}}{\partial \lambda} = 0$, which gives

$$\boldsymbol{\Sigma} \mathbf{v}_1 = \lambda \mathbf{v}_1, \quad \mathbf{v}_1^\top \mathbf{v}_1 = 1. \qquad (2\text{-}8)$$

Since $\mathbf{v}_1^\top \mathbf{v}_1 = 1$ is the same as the constraint function (2-6), the equation to be solved is $\boldsymbol{\Sigma} \mathbf{v}_1 = \lambda \mathbf{v}_1$. Here, $\boldsymbol{\Sigma} \mathbf{v}_1 = \lambda \mathbf{v}_1$ coincides with the definition of eigen decomposition, and it can be algebraically manipulated as follows.

$$\begin{aligned} \boldsymbol{\Sigma} \mathbf{v}_1 &= \lambda \mathbf{v}_1 \\ \mathbf{v}_1^\top \boldsymbol{\Sigma} \mathbf{v}_1 &= \mathbf{v}_1^\top \lambda \mathbf{v}_1 \\ \mathbf{v}_1^\top \boldsymbol{\Sigma} \mathbf{v}_1 &= \lambda \mathbf{v}_1^\top \mathbf{v}_1 \end{aligned}$$

$$\mathbf{v}_1^\top \boldsymbol{\Sigma} \mathbf{v}_1 = \lambda \qquad (2\text{-}9)$$

From equation (2-5) and equation (2-9), it can be seen that λ corresponds to the variance of the projected data $\mathbf{z}$. In other words, finding the basis vector $\mathbf{v}_1$ that maximizes $\mathrm{Var}(\mathbf{z})$ is equivalent to finding the eigenvector corresponding to the largest eigenvalue of the covariance matrix of A'.

We have considered the case of reducing the dimensionality to one dimension using a single basis vector. Now, we consider the general case of reducing the dimensionality to p

dimensions using p basis vectors (with $p \in \mathbb{N}, 1 \le p \le m$). We denote the p-th basis vector by $\mathbf{v}_p$. To eliminate multicollinearity, it is sufficient to require that $\mathbf{v}_p$ be orthogonal to $\mathbf{v}_i$ for all $1 \le i \le p-1$. Thus, the following constraint is imposed:

$$g_i(\mathbf{v}_p) = \mathbf{v}_i^\top \mathbf{v}_p = 0 \quad \text{for } i = 1, 2, \ldots, p-1. \tag{2-10}$$

By incorporating the following functions (2-11) and (2-12), which generalize equations (2-5) and (2-6), the Lagrangian function can be defined by (2-13).

$$\max \mathrm{Var}(\mathbf{z}_p) = \max \mathbf{v}_p^\top \mathbf{\Sigma} \mathbf{v}_p \tag{2-11}$$

$$g(\mathbf{v}_p) = \mathbf{v}_p^\top \mathbf{v}_p - 1 = 0 \tag{2-12}$$

$$\mathcal{L}(\mathbf{v}_p, \lambda_p, \mu) = \mathbf{v}_p^\top \mathbf{\Sigma} \mathbf{v}_p - \lambda_p \left(\mathbf{v}_p^\top \mathbf{v}_p - 1 \right) - \sum_{i=1}^{p-1} \mu_i \mathbf{v}_i^\top \mathbf{v}_p \tag{2-13}$$

By the method of Lagrange multipliers, when maximizing $\mathbf{v}_p^\top \mathbf{\Sigma} \mathbf{v}_p$, the following conditions are satisfied:

$$\frac{\partial \mathcal{L}}{\partial \mathbf{v}_p} = 0 \Leftrightarrow 2\mathbf{\Sigma} \mathbf{v}_p - 2\lambda_p \mathbf{v}_p - \sum_{i=1}^{p-1} \mu_i \mathbf{v}_i = 0$$

$$\therefore \mathbf{\Sigma} \mathbf{v}_p = \lambda_p \mathbf{v}_p + \frac{1}{2} \sum_{i=1}^{p-1} \mu_i \mathbf{v}_i \tag{2-14}$$

$$\frac{\partial \mathcal{L}}{\partial \lambda_p} = 0 \Leftrightarrow \mathbf{v}_p^\top \mathbf{v}_p - 1 = 0 \tag{2-15}$$

$$\frac{\partial \mathcal{L}}{\partial \mu_i} = 0 \Leftrightarrow \mathbf{v}_i^\top \mathbf{v}_p = 0 \quad \text{for } i = 1, 2, \ldots, p-1 \tag{2-16}$$

Since (2-15) and (2-16) are same with (2-12) and (2-10), we only need to consider (2-14). Multiplying both sides of (2-14) by $\mathbf{v}_\ell^\top \quad (\ell = 1, 2, \ldots, p-1)$ yields

$$\mathbf{v}_\ell^\top \boldsymbol{\Sigma} \mathbf{v}_p = \mathbf{v}_\ell^\top \left(\lambda_p \mathbf{v}_p + \frac{1}{2}\sum_{i=1}^{p-1} \mu_i \mathbf{v}_i \right). \tag{2-17}$$

Because $\boldsymbol{\Sigma}$ is symmetric (i.e., $\boldsymbol{\Sigma} = \boldsymbol{\Sigma}^T$) and given the constraint

$\mathbf{v}_i^\top \mathbf{v}_p = 0$ for $i = 1, 2, \ldots, p-1$, the left-hand side simplifies to

$$\mathbf{v}_\ell^\top \boldsymbol{\Sigma} \mathbf{v}_p = (\boldsymbol{\Sigma}^\top \mathbf{v}_\ell)^\top \mathbf{v}_p = (\boldsymbol{\Sigma} \mathbf{v}_\ell)^\top \mathbf{v}_p = \boldsymbol{\Sigma} \mathbf{v}_\ell^\top \mathbf{v}_p = 0. \tag{2-18}$$

Next, the right-hand side is

$$\begin{aligned} \mathbf{v}_\ell^\top \left(\lambda_p \mathbf{v}_p + \frac{1}{2}\sum_{i=1}^{p-1} \mu_i \mathbf{v}_i \right) &= \mathbf{v}_\ell^\top \lambda_p \mathbf{v}_p + \frac{1}{2} \mathbf{v}_\ell^\top \sum_{i=1}^{p-1} \mu_i \mathbf{v}_i \\ &= \lambda_p \mathbf{v}_\ell^\top \mathbf{v}_p + \frac{1}{2}\sum_{i=1}^{p-1} \mu_i \mathbf{v}_\ell^\top \mathbf{v}_i \\ &= 0 + \frac{1}{2}\sum_{i=1}^{p-1} \mu_i \mathbf{v}_\ell^\top \mathbf{v}_i. \end{aligned} \tag{2-19}$$

Here, since

$$\mathbf{v}_i^\top \mathbf{v}_j = \begin{cases} 1 & \text{if } i = j, \\ 0 & \text{if } i \neq j, \end{cases} \tag{2-20}$$

(2-19) can be further transformed as follows.

$$\frac{1}{2}\sum_{i=1}^{p-1} \mu_i \mathbf{v}_\ell^\top \mathbf{v}_i = \frac{1}{2}\mu_\ell \tag{2-21}$$

Since (2-18) and (2-21), equation (2-17) can be transformed as follows.

$$\begin{gathered} \mathbf{v}_\ell^\top \boldsymbol{\Sigma} \mathbf{v}_p = \mathbf{v}_\ell^\top \left(\lambda_p \mathbf{v}_p + \frac{1}{2}\sum_{i=1}^{p-1} \mu_i \mathbf{v}_i \right) \Leftrightarrow 0 = \frac{1}{2}\mu_\ell \\ \therefore \mu_\ell = 0 \end{gathered} \tag{2-22}$$

Therefore, equation (2-14) can be transformed as follows.

$$\boldsymbol{\Sigma} \mathbf{v}_p = \lambda \mathbf{v}_p + \frac{1}{2}\sum_{i=1}^{p-1} \mu_i \mathbf{v}_i \Leftrightarrow \boldsymbol{\Sigma} \mathbf{v}_p = \lambda \mathbf{v}_p \tag{2-23}$$

Because equation (2-23) is the definition of eigen decomposition, the calculation of the p-th basis vector $\mathbf{v}_p$ also reduces to the eigen decomposition of the covariance matrix of A'. Since λ corresponds to the variance of the projected data, when reducing to p dimensions, the p eigenvectors corresponding to the largest eigenvalues are selected to span the space. The data projected onto these eigenvectors, denoted by $\mathbf{z}_p$, are collected into $Z = (\mathbf{z}_1 \quad \mathbf{z}_2 \quad \dots \quad \mathbf{z}_p)$, which represents the dimensionally reduced data. Taking into account the unique aspect of blending commercial media, we have implemented the approach described above to reduce the dimensionality to a vector space that is free from multicollinearity while preserving as much variance as possible in the media compositions. Here, this procedure—the process of "repeated orthogonal projection onto the axis of maximum variance"—corresponds exactly to the operation performed by PCA. In PCA, the covariance matrix Σ is decomposed via eigen decomposition, and the principal components $\mathbf{v}_1, \mathbf{v}_2, \dots, \mathbf{v}_p$ are selected sequentially from the eigenvectors corresponding to the largest eigenvalues, mapping the original data A into a p-dimensional space according to

$$Z = AV_p, \quad V_p = [\mathbf{v}_1, \mathbf{v}_2, \dots, \mathbf{v}_p]. \tag{2-24}$$

Therefore, although several methods might be considered if one only focuses on reducing the dimensionality to a vector space free of multicollinearity, under the goal of retaining as much variance in the media composition data as possible, PCA is the most appropriate approach in the experimental design of media blending.

Supplemental Tables

Supplemental Table S1 Analytical conditions for the measurement of metal ions using ICPMS-2030

The condition name	The value
Radio frequency power [kW]	1.2
Flow rate of plasma gas [L/min]	9.0
Flow rate of auxiliary gas [L/min]	1.1
Flow rate of carrier gas [L/min]	0.7
Pump speed [rpm]	20
Cell gas	Helium

Supplemental Table S2 Details of model construction according to a guideline for supervised learning approaches in biological studies.

Broad topic	Questions	Answers
Data	What is the source of the data?	It is a direct experiment.
	How many real samples are there?	There are 360 samples (120 conditions, 3 biological replicates).
	Has the dataset been previously used by other papers?	No.
	How many data points are in the training and test sets?	Training: 216, Test: 144 (Training: Test = 6 : 4).
	Are the distributions of the training and test sets same?	Yes.<br>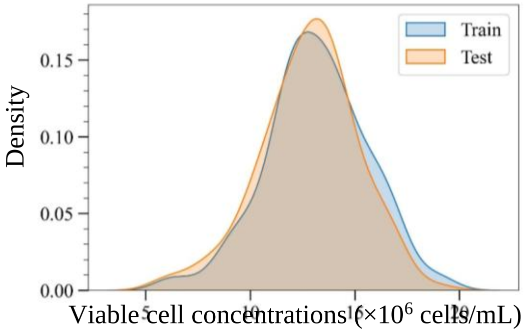
	How were the sets split? Are the training and test sets independent?	Yes, the training set and test set are independent. To ensure that data with the same condition are consistently grouped and exclusively splitted to either the training set or the test set, we employed GroupKFold function in scikit-learn for Python.
	What preprocessing steps were performed?	The training set was standardized to mean 0 and standard deviation 1. The standardization function was applied to the test set.
Optimization	What is the ML algorithm class used? Is the ML algorithm new?	Not new. We selected the algorithm based on the following assumption for the medium components.<br>1. For drastic changes: RF, GBDT, XGBoost.<br>2. For multimodal behavior: Nonlinear SVR, Nonlinear GPR.<br>3. For linear explanation: PLS-R, Ridge, Lasso, ElasticNet, Linear SVR, Linear GPR.
	Were any overfitting prevention techniques used?	Yes.<br>1. For hyperparameter tuning, the training set was divided using 5-fold cross-validation, and the hyperparameters corresponding to the highest value of (mean - standard deviation) of the predictive error (R^2) on the validation set were selected.<br>2. In this cross-validation as well, data with the same condition were consistently grouped and exclusively assigned to either the training set or the validation set.<br>3. In particular, for the hyperparameters of the non-linear models, the optimization was conducted within ranges that avoided excessive model complexity.

(continued)

Table S2 (continued)

Broad topic	Questions	Answers
Model	Is the model black box or interpretable?	Given the presence of black-box models, we calculated variable importances by employing an adjusted Permutation Feature Importance (PFI), which facilitates a uniform assessment of interpretability across any model.
Evaluation	How was the method evaluated (for example cross-validation, independent dataset)?	Independent test set was used. The optimized hyperparameters were used to retrain the entire training set, and then test set was predicted using the trained model.
	Which performance metrics are reported?	R^2 and Mean Square Error (MSE) were used as performance metrics.<br><br>R^2 is defined as: $R^2 = 1 - \frac{\sum_{i=1}^{n}(y_i - \hat{y}_i)^2}{\sum_{i=1}^{n}(y_i - \bar{y})^2}$.<br><br>R^2 was used to evaluate the extent to which the model's predictions explain the variance of the target variable *y*.<br><br>MSE is defined as: $\mathrm{MSE} = \frac{1}{n}\sum_{i=1}^{n}(y_i - \hat{y}_i)^2$.<br><br>MSE was adopted to evaluate whether the model has large errors, owing to its sensitivity to outliers.
	Was a comparison to simpler baselines performed?	Yes, we evaluated the improvement in predictive accuracy relative to a baseline model that predicts the mean value.
	Are the results statistically significant to claim that the method is superior to baselines?	Yes, test set was randomly splitted into five sets, and MSE was computed for each set. Paired t-test was performed between baseline model and other models. *p*-values were adjusted using Holm correction, and a one-sided test with $\alpha = 0.05$ was performed to assess the significance relative to the baseline model.
Availability	Are the data and the code used in the study available?	Yes, we have uploaded the files to GitHub (https://github.com/HK-bio/medium_study_repo/tree/main/paper_202504). This work is licensed under the Creative Commons Attribution-NonCommercial-ShareAlike 4.0 International License.

Supplemental Table S3 Top 12 components of variable importance in projection (VIP) score in PLS-R model and adjusted permutation feature importance (PFI)

	**VIP in PLS-R**	PFI in PLS-R	PFI in all four superior models[*1]
**The top 12 components**	Asparagine	Asparagine	Asparagine
	Cu	Tyrosine	Tyrosine
	Glucose	Glucose	Glucose
	Tyrosine	Choline	Choline
	Choline	Histidine	Histidine
	Hypoxanthine	Leucine	Leucine
	Leucine	Zn	Zn
	Histidine	Lysine	Valine
	5-Oxoproline	Isoleucine	Lysine
	Methionine	Valine	5-Oxoproline
	2-Aminoethanol	Cystine	Cu
	Valine	Gluconic acid	Methionine

***1** PFI of the top variables commonly identified in the four superior models

Supplemental Figures

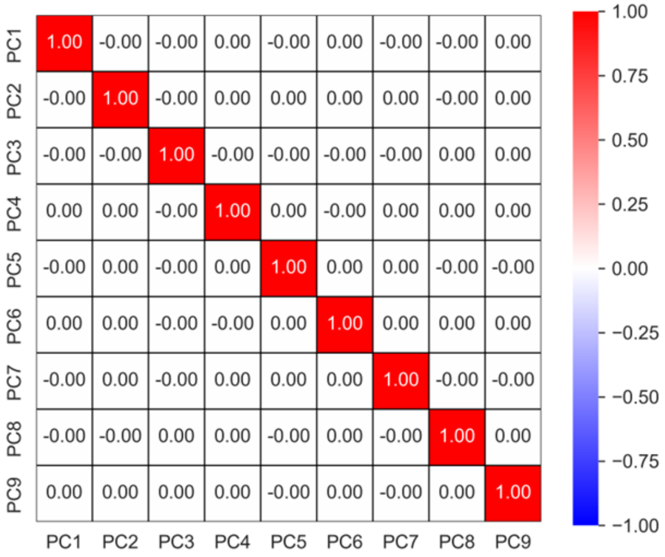

Supplemental Fig. S1 Heatmap of the pearson's product-moment correlation coefficients between principal components of the medium components in 11 types of chemically-defined media. Although theoretically obvious, the correlations between all principal components were zero. PC, principal components

a

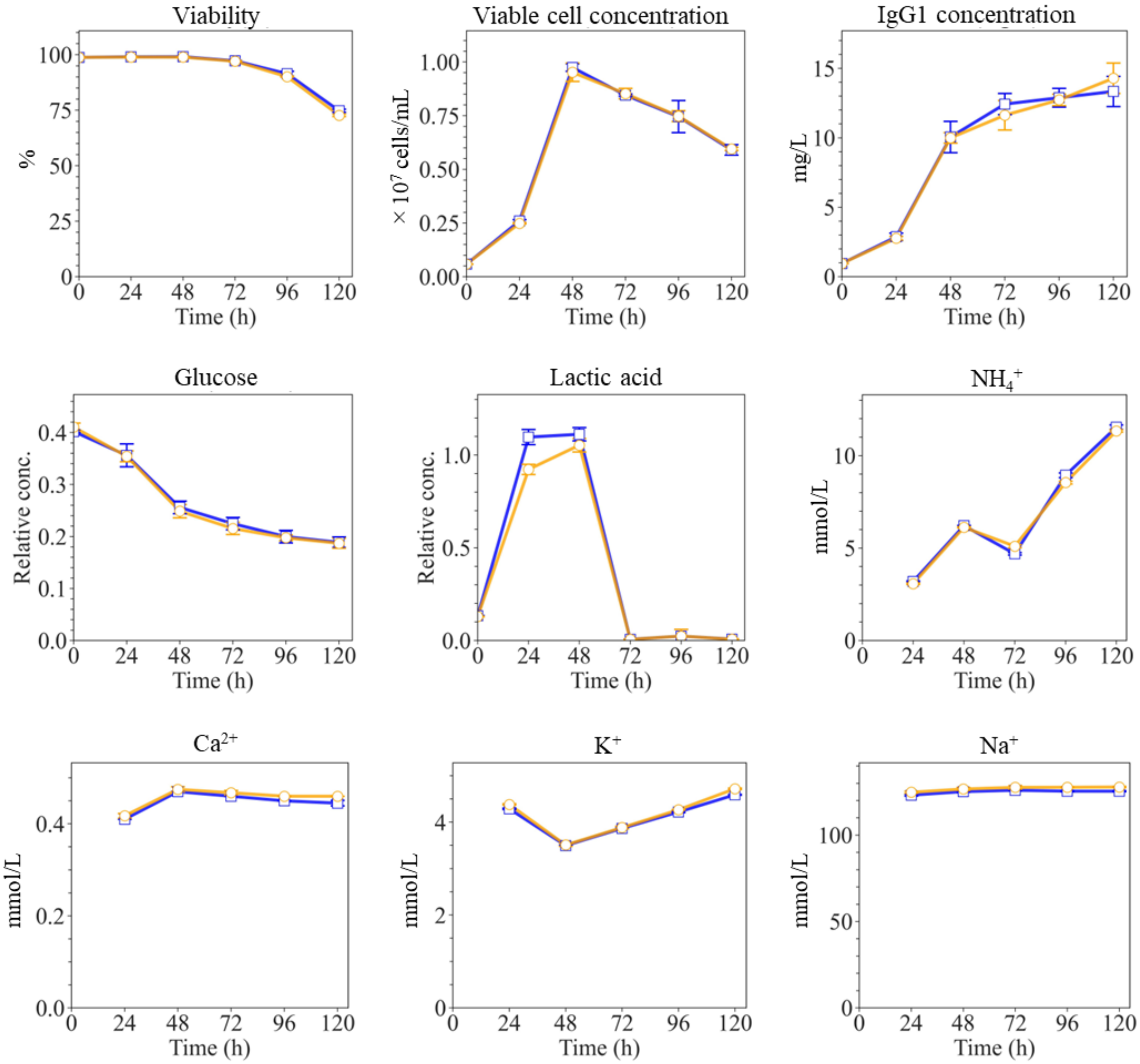

Viability
%
100
75
50
25
0
0 24 48 72 96 120
Time (h)
Viable cell concentration
× 10^7 cells/mL
1.00
0.75
0.50
0.25
0.00
0 24 48 72 96 120
Time (h)
IgG1 concentration
mg/L
15
10
5
0
0 24 48 72 96 120
Time (h)
Glucose
Relative conc.
0.4
0.3
0.2
0.1
0.0
0 24 48 72 96 120
Time (h)
Lactic acid
Relative conc.
1.0
0.5
0.0
0 24 48 72 96 120
Time (h)
NH4+
mmol/L
10
5
0
0 24 48 72 96 120
Time (h)
Ca2+
mmol/L
0.4
0.2
0.0
0 24 48 72 96 120
Time (h)
K+
mmol/L
4
2
0
0 24 48 72 96 120
Time (h)
Na+
mmol/L
100
50
0
0 24 48 72 96 120
Time (h)

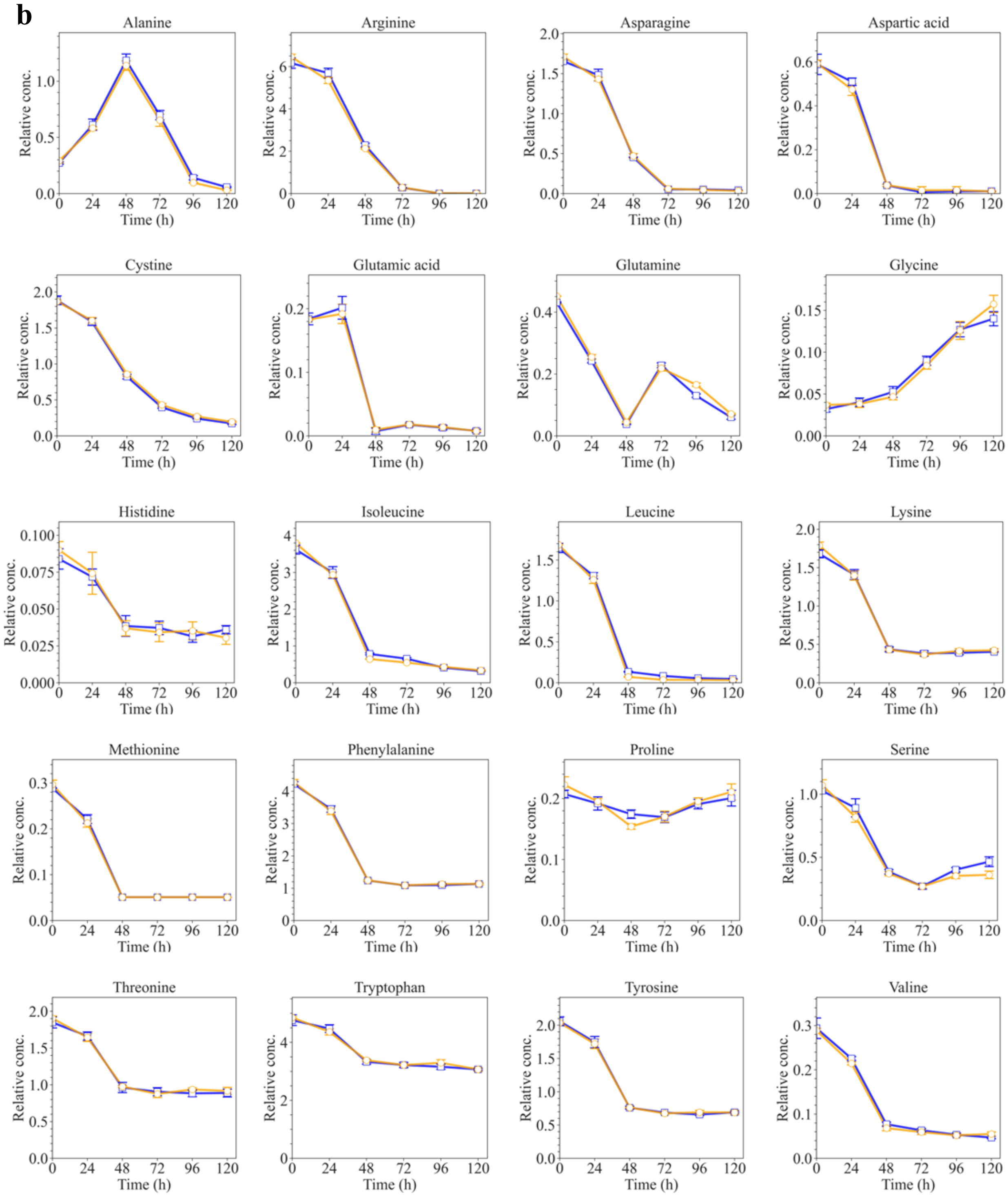

Supplemental Fig. S2 Comparison of the culture results of CHL-YN between 24 well plate and flask. Batch cultures were carried out with biological replicates (*n* = 4). Time course of **a** viability, viable cell concentrations, IgG1 concentration, major process parameters, **b** and amino acids in supernatants. Orange circles represent the results using 24-well plate, and blue squares

represent the results using flask. The culture results using 24-well plates were comparable to those obtained using flasks across all culture parameters. Relative conc., Relative concentration
